# Status and trends of mercury pollution of the atmosphere and terrestrial ecosystems in Poland

**DOI:** 10.1007/s13280-021-01505-1

**Published:** 2021-03-23

**Authors:** Agnieszka Jędruch, Lucyna Falkowska, Dominika Saniewska, Maciej Durkalec, Agnieszka Nawrocka, Elżbieta Kalisińska, Artur Kowalski, Józef M. Pacyna

**Affiliations:** 1 Faculty of Oceanography and Geography, Institute of Oceanography, Department of Marine Chemistry and Environmental Protection, University of Gdańsk, Al. Marszałka Piłsudskiego 46, 81-378 Gdynia, Poland; 2 Department of Pharmacology and Toxicology, National Veterinary Research Institute, Al. Partyzantów 57, 24-100 Puławy, Poland; 3 Faculty of Pharmacy, Medical Biotechnology and Laboratory Medicine, Department of Biology and Medical Parasitology, Pomeranian Medical University, Al. Powstańców Wielkopolskich 72, 70-111 Szczecin, Poland; 4 Faculty of Chemistry, Department of Analytical and Environmental Chemistry, Adam Mickiewicz University, ul. Uniwersytetu Poznańskiego 8, 61-614 Poznań, Poland; 5 Faculty of Energy and Fuels, AGH University of Science and Technology, ul. Mickiewicza 30, 30-059 Kraków, Poland

**Keywords:** Animals, Atmosphere, Bioindicators, Mercury, Plants, Temporal trends

## Abstract

The goal of this paper is to assess the current status and trends of total mercury (THg) contamination of the atmosphere and terrestrial ecosystems in Poland. The study shows that the reduced domestic and worldwide atmospheric emission of Hg resulted in decreased THg level in the terrestrial biotope and biosphere. Considering that Poland is one of the main Hg emitters in Europe, the THg concentrations in its abiotic environment are still elevated. However, the THg level in terrestrial organisms is relatively low, which is because a large proportion of Hg deposited on land is accumulated in organic-rich soils. Regarding the THg concentration, consumption of wildlife and livestock from Poland is safe for humans. Nevertheless, the authors indicate the need for effective environmental monitoring, based on selected bioindicators, which is crucial considering the slowing reduction of Hg emission combined with the consequences of the changing climate.

**Supplementary Information:**

The online version contains supplementary material available at 10.1007/s13280-021-01505-1.

## Introduction

Mercury (Hg) is a contaminant introduced into the environment from natural sources, including volcanoes and forest fires, re-emission from the ocean and terrestrial surfaces, and anthropogenic activities in the energy production, metallurgy, waste incineration, and other industrial processes. The re-emission of Hg into the atmosphere plays particularly important role in the Hg cycle, however, a portion of Hg circulating currently through the environment media and released years ago, is very difficult to determine (HELCOM 2018; UNEP 2019). It was estimated that, as a result of anthropogenic activity over the past century, the Hg concentration in the atmosphere has increased by 3 to 5 times and tripled in the surface ocean water (UNEP 2019). Human-caused emission significantly increased the global Hg pool in the environment and resulted in its elevated concentration in air, water, soil and sediments, often exceeding the geochemical background level (Pacyna et al. 2010; Pirrone et al. 2010; Bishop et al. 2020). For several decades, numerous actions have been taken to reduce the anthropogenic Hg emission and to prevent its spread in the environment (Pacyna et al. 2010; EMEP 2016). The Minamata Convention (UNEP 2013), creating a legal framework to protect human health and the environment from the adverse effects of Hg, sets out the direction of necessary actions for efficient control of the environment and its protection for future generations. The European Union (EU) Member States have committed themselves to conduct long-term policy and international cooperation to reduce Hg emissions and subsequent deposition in the environment (UNEP 2013, 2019; EC 2017). The Minamata Convention is a significant challenge for Poland, a country with some of the highest Hg emissions and depositions in Europe (in 2016 Poland accounted for 18% of total atmospheric Hg emission in the EU) (EEA 2018). Nevertheless, Poland took actions leading to a substantial reduction in Hg emission at the end of the twentieth century thereby contributing to a decrease in the Hg deposition (Pacyna et al. 2010; Zhang et al. 2016; HELCOM 2018; KOBiZE 2019).

The current model-based estimates of Hg inflow (EMEP 2016, 2018; HELCOM 2018) suggest that the biotope and biosphere in Central Europe may be more exposed to Hg than other regions. This is related to the industrialisation of this area, especially the carbon-based energy economy of its countries, which is a major source of Hg. Therefore, a primary goal of this paper is to investigate the exposure to Hg of terrestrial flora and fauna from Poland. We also aim to determine not only the current status of total Hg (THg) in biota but also to indicate time trends of THg level and factors conditioning them. We hypothesise that the decrease of THg concentration in the terrestrial food webs results from a reduction in anthropogenic emission and thus deposition of Hg. However, ongoing climate change and other environmental pressures may affect this tendency. Consequently, the pool of bioavailable Hg and its tropic transfer may both increase.

## Materials and Methods

In Poland, the first results on Hg level, both in the case of terrestrial (i.e. Szprengier 1976; Kossakowski 1979) and freshwater ecosystems (i.e. Nabrzyski 1975; Gajewska and Nabrzyski 1977), date back to the 1970s. Although methylmercury (MeHg) is one of the most poisonous among Hg compounds, this study focuses only on THg. This is because the knowledge of Hg speciation, both in abiotic and biotic environmental compartments in Poland, is mostly based on very limited data. To determine the status and the temporal trends of THg pollution in Poland, a total of 85 data sources were used in this work, while the discussion of the results was based on additional 163 literature sources (Fig. S1). Due to the arrangement of the article, some of these references are provided in Electronic Supplementary Material (ESM). Besides information on data collection, the ESM also contains details of results analysis.

## Discussion

### Hg level in abiotic environmental compartments

The land surface contributes to a complex bi-directional Hg exchange with the atmosphere. Hg deposited on land enters the soil that constitutes the largest pool of Hg in the terrestrial environment. Surface runoff and soil erosion are a major source of Hg that accumulates in biota from aquatic ecosystems (Kowalski et al. 2007; Jędruch et al. 2019; Bishop et al. 2020). In this part, we summarise the findings on THg level in the key abiotic components of the terrestrial environment, namely atmosphere (Sect. Atmosphere), soil (Sect. Soil), and freshwater bodies (Sect. Waterbodies), conducted in Poland.

#### Atmosphere

The main sources of anthropogenic Hg are processes involving high temperatures, including the burning of fossil fuels, the smelting of non-ferrous metals, iron and steel foundries, as well as the cement industry and gold production. In Poland, 71% of Hg is emitted from the energy sector, while the industrial processes and product use are responsible for 26% of total national Hg emission (KOBiZE 2019). However, it is estimated that only about half of the atmospheric Hg in Poland originates from its territory. The remaining Hg pool is transported mainly from Western European countries along with air masses travelling from the south-west of the continent (Petersen 1999). The most important Hg sources to the atmosphere in Poland are located in industrial regions. In Silesia region (southern Poland), which is one of the European air pollution ‘hot-spots’, the Hg emission was 3.5 Mg in 2005, while in the less industrialised regions (western and northern Poland) it amounted to 0.2 Mg (Hławiczka 2008). Due to the use of coal as the primary energy source in Poland, individual home furnaces are seen as the major cause of local or dispersed air pollution.

Hg is emitted into the atmosphere mainly in gaseous elemental form and can remain there for 12–18 months (Holmes et al. 2006). In 2017, the average concentration of total gaseous mercury (TGM) measured at non-urban stations (1.5 ng m^−3^) was 25% higher than the European average TGM concentration (1.2 ng m^−3^) (Skotak et al. 2019). TGM values measured in Poland were similar to those observed in other industrialised countries, such as Germany (1.5 ng m^−3^) and UK (1.4 ng m^−3^). The high average TGM level in Poland was affected by the results obtained at the stations located in the polluted southern part of the country, where the annual TGM concentrations were 1.7 ng m^−3^ or higher (Skotak et al. 2019). A similar trend was also observed for the total particulate mercury (TPM). In northern Poland, the TPM concentrations (Korejwo et al. 2020) were similar to those measured in the Swedish coastal zone (around 10 pg m^−3^) (Wängberg et al. 2001), while in southern Poland Hg concentration in the particulate matter oscillated around 30 pg m^−3^ (Pyta et al. 2020). TGM can be oxidised to Hg^2+^, which is much more reactive, hence it is removed from the atmosphere with dry and wet deposition. A dry deposition usually accounts for less than 30% of the total atmospheric Hg inflow, which was noted in Poland (Saniewska et al. 2014a; Bełdowska et al. 2016), as well as in other regions of the Baltic area (www.ebas.nilu.no). The Hg deposition in Poland fluctuates spatially. In northern Poland it varies from 10 to 15 g km^−2^ year^−1^ and is similar to the Hg deposition flux over the southern Baltic Sea. In central Poland, atmospheric deposition of Hg ranges from 15 to 30 g km^−2^ year^−1^, while the industrialised south of the country receives over 30 g km^−2^ year^−1^ (EMEP, 2016).

**Fig. 1:**
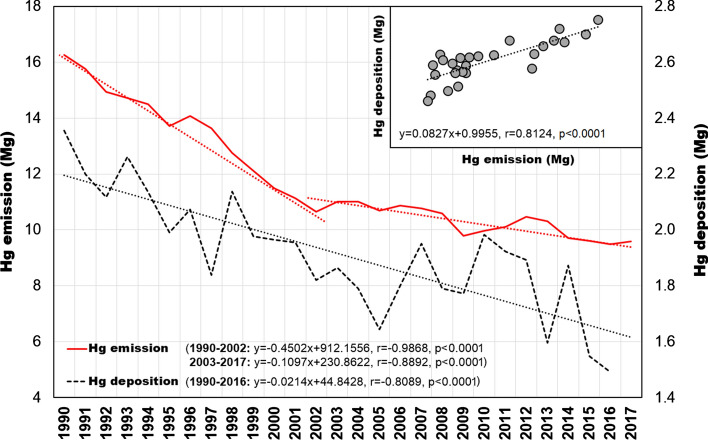
Long-term changes in annual total Hg emission (Mg) and deposition (Mg) in Poland and southern Baltic Sea (EMEP 2018; KOBiZE, 2019)

In 1983–1989 the estimated emission of Hg from Polish territory exceeded 20 Mg year^−1^ and increased on average by 0.27 Mg year^−1^ (Pirrone et al. 1996). The reduction of Hg emission in Poland took place in 1990–2002 and amounted to − 0.43 Mg year^−1^. The stabilisation period for Hg emissions occurred in the years 2003–2017 with a weak trend of − 0.1 Mg year^−1^. The latest data show that the national emission of Hg does not exceed 10 Mg year^−1^ (Fig. S2). However, it should be noted that the decrease in Hg emission in Poland (by 42% since 1990) is one of the smallest among European countries reported to the Convention on Long-range Transboundary Air Pollution (LRTAP). To compare, during this period, Hg emissions in Germany and Sweden were reduced by 74% and in the UK by 90% (EEA 2019). Along with long-term changes in Hg emissions from the territory of Poland, there were simultaneous changes in its deposition (Fig. 1). The strongest decreasing trend by 30–40% in Hg concentration in the air noted in the northern hemisphere in the 1990s resulted from the intentional withdrawal of Hg from commercial products and control technologies. For the 1990–2012 period, Zhang et al. (2016) determined a decrease in Hg^0^ concentration in the atmosphere and its deposition at − 2.1% year^−1^ and − 2.2% year^−1^, respectively. There is no doubt that Hg emission forecasts indicate its slow reduction, but global emission models are not as optimistic, depending on the adopted scenario (Rafaj et al. 2013; UNEP 2013). According to emission control strategy, the decrease in Hg concentration in remote industrial areas may amount to 15–20% of the present level. Maintaining the emission status quo in industrialised regions can contribute to an increase in Hg levels of 2 to 25%, and 1.5 to 5% away from these regions. Even a small increase in anthropogenic emissions or re-emission from Hg accumulation areas may cause significant emission growth.

#### Soil

**Fig. 2:**
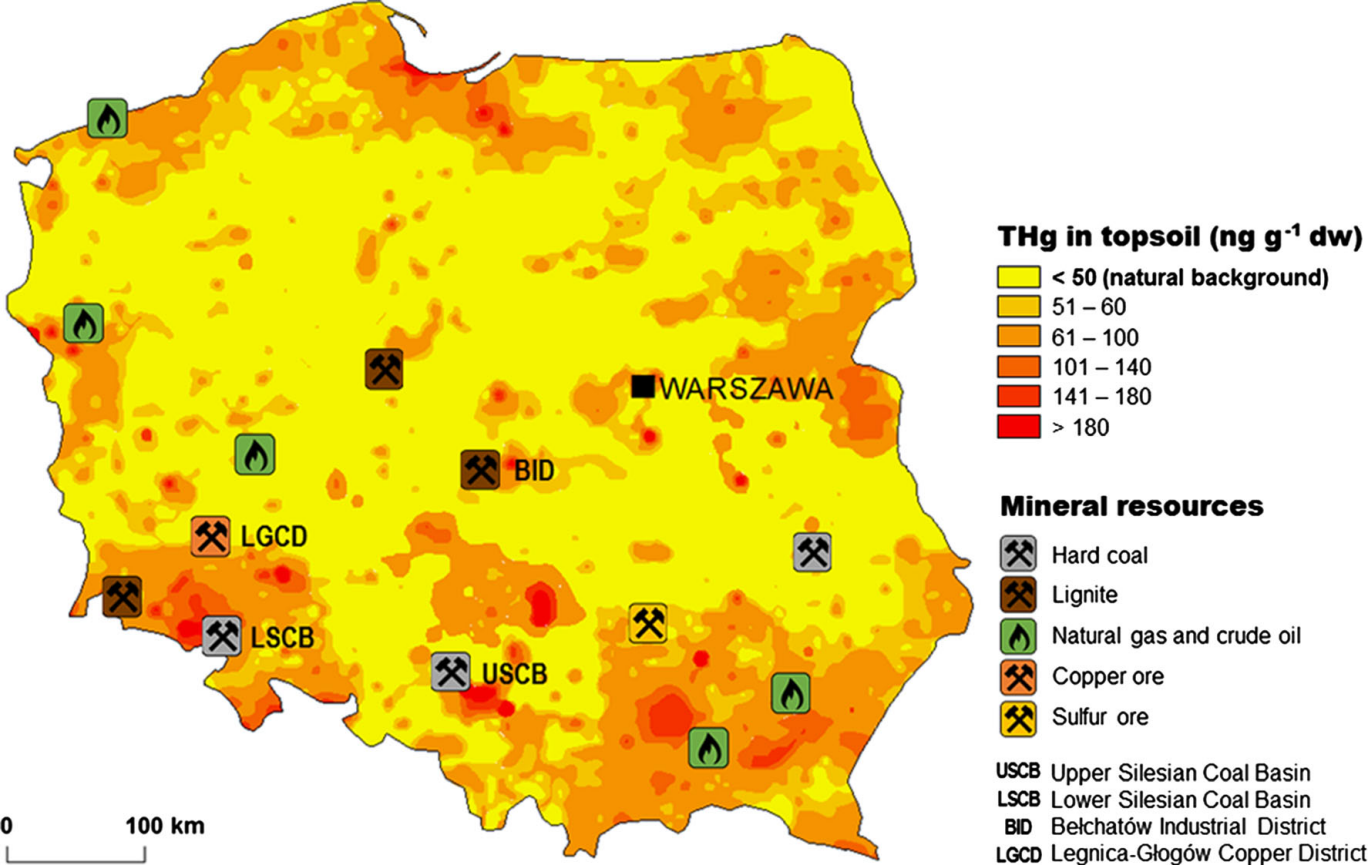
Total Hg (THg) concentration (ng g^−1^ dw) in the topsoil (0–20 cm) in Poland (based on data published by Pasieczna (2012)) in 1991–1992 together with the locations of main mineral resources and industrial districts

**Fig. 3:**
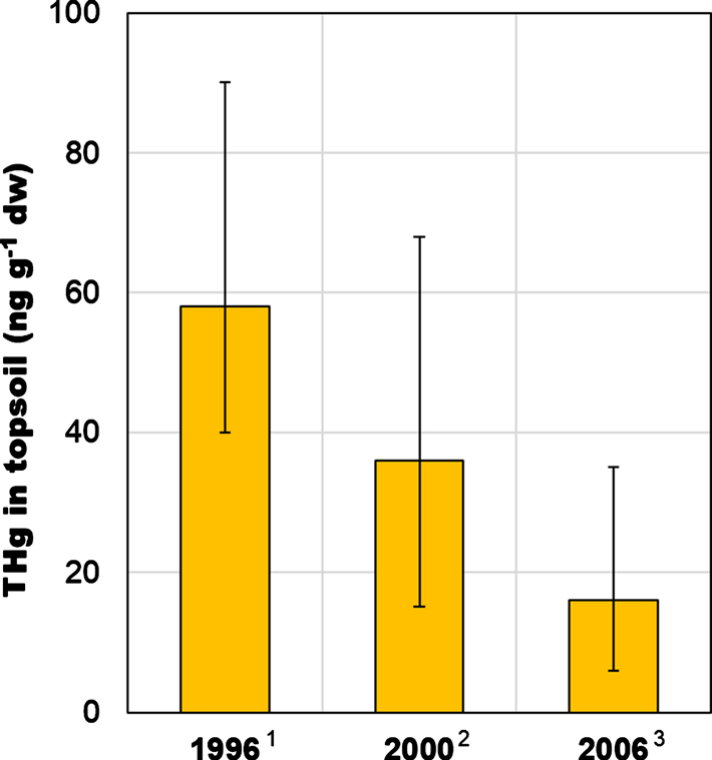
Total Hg (THg) concentration (ng g^−1^ dw) (mean and range) in the topsoil of northern Poland (Tri-City Landscape Park) (^1^Falandysz et al. 2003; ^2^Falandysz and Bielawski 2007; ^3^Saba et al. 2016)

Hg concentration in soil is related to its geological structure and human activity in a given area. The average THg content in Polish soils usually did not exceed 50 ng g^−1^ dw (dry weight), which is considered as the geochemical background (Pasieczna 2012). The average THg concentration in soils from non-built-up areas was 17 ng g^−1^ dw which is close to the mean concentration in unpolluted European soils. In southern Poland, higher THg concentration in soil (over 60 ng g^−1^ dw) was associated with the elevated Hg level in the bedrock (FOREGS 2005) (Fig. 2). In general, the THg content in urban soils was 2 to 4 times higher than in background areas (Pasieczna 2012). Research conducted in northern Poland has shown that over 70% of THg in soil occurred in the soluble labile fraction (i.e. Hg halides, Hg bound to humic acids or MeHg), while stable and insoluble forms (i.e. HgS or matrix-bound Hg) accounted for less than 30% (Gębka et al. 2020). Similar results were obtained in contaminated soil from southern Poland, where about 35% of soil Hg was in stable form (Pogrzeba et al. 2016). During the twentieth and twenty-first century THg concentration in soils of the industrialised and urbanised areas of northern Poland decreased threefold (Fig. 3), which was probably a response to the reduction of Hg emissions and deposition that took place in Poland in the 1990s (Fig. 1).

The outflow of Hg from soils into rivers depends on the retention capacity of the catchment area. In natural regions, only 10–30% of Hg deposited from the atmosphere flows from the catchment area to the river, while in the human-transformed catchment the Hg outflow can reach 65% (Driscoll et al. 1998). Research by Saniewska et al. (2014b, 2019) showed that in transformed areas, water in rivers was several times more contaminated with THg compared to natural areas. This is important as the agricultural areas cover two-thirds of Poland, which stimulates such a great Hg outflow from soil to rivers and then to the sea. As shown by Driscoll et al. (1998) over 70% of Hg deposited on land retains in the soil and due to atmospheric deposition, this pool is successively supplemented. This means that soil is not only storage but also an important Hg source to the aquatic environment and will remain so for several thousands of years (Larssen et al. 2008).

#### Waterbodies

Hg enters the freshwater ecosystem mainly through outflow from soils. The Hg level in water is affected mainly by the concentration of dissolved organic matter, which is a link between land and the aquatic environment. It is also influenced by physicochemical parameters, land cover or watershed disturbances (Bravo et al. 2018; Bishop et al. 2020). In comparison to soils, the level of Hg in the water of rivers and lakes in Poland is understudied, which is mainly due to the methodological difficulties of Hg analysis in liquid samples versus solid matrices. Although the THg concentration in rivers is routinely monitored, owing the weaknesses of the analytical methods used, such as limits of quantification often exceeding the THg level in the water (HELCOM 2018), the number of reliable data are low. The THg concentration in the unfiltered water of Vistula, the largest river in Poland and the second largest river discharging into the Baltic Sea, averaged to 6.3 ng L^−1^ and the ranged from 4.7 to 11.7 ng L^−1^ during the year (Saniewska et al. 2014c) (Table S1a). These concentrations met the EU environmental quality standards (EQS) for THg concentration in inland waters (EC 2008), moreover none of the values exceeded 12 ng L^−1^, above which the chronic toxic effects on aquatic organisms is observed (US EPA 1992). In small watercourses in northern Poland, the THg concentration was similar to the Vistula and ranged from 4.1 to 6.7 ng L^−1^ (Gębka et al. 2018). The highest THg concentration in rivers occurred during thaws, which released pollutants that had been accumulating over the winter (Gębka et al. 2020). Downpours and floods had a crucial impact on a THg load carried by rivers (Saniewska et al. 2014c; Jędruch et al. 2017) (Table S1b). During these periods, the daily outflow of THg from soil was estimated at 90–150% of the daily wet deposition flux (Saniewska et al. 2018).

The results of a large-scale study conducted in streams across Europe by Bravo et al. (2018), showed that the measured THg concentrations were lower than in Polish rivers, and ranged from 0.1 to 2.8 ng L^−1^ (Table S1a). These differences result mainly from the type of catchment area, i.e. streams enriched in terrestrial dissolved organic matter revealed the elevated total Hg concentrations, as in the case of Sweden or UK (Bravo et al. 2018). This may therefore indicate that one of the factors influencing higher THg concentrations in Polish rivers is the agriculture‐dominated catchment. Moreover, the THg concentrations in Poland were similar to values measured in the agricultural watercourses in Sweden (2.4–4.5 ng L^−1^) (Eklöf et al. 2012). Unfortunately, there are no data on the MeHg concentration in the riverine water in Poland. Considering the share of MeHg in THg in the European rivers varied widely from 1 to 43% (Bravo et al. 2018), it is hard to estimate its percentage in Polish watercourses.

The THg concentration in waters of lakes and ponds in western Poland was several times higher compared to rivers and ranged from 8 to 24 ng L^−1^ and 16 to 31 ng L^−1^, respectively (Kowalski et al. 2007). In lakes considered as polluted, Hg concentration in the water exceeded 100 ng L^−1^ (Baralkiewicz et al. 2006). This may be of environmental importance considering that the share of toxic MeHg in water was relatively high and amounted to about 10% of THg, which is consistent with the results of studies carried out in boreal and subarctic lakes (Emmerton et al. 2018). The results indicate that Polish lakes are more contaminated with THg than lakes from southern Scandinavia (Lindström 2001; Braaten et al. 2014). However, the available data are very local and do not reflect the scale of the THg pollution of lakes in Poland.

### Hg level in terrestrial plants and animals

Most studies on Hg level in organisms have focused on the aquatic environment, and the role of Hg accumulation in terrestrial food webs is neglected. A clear preponderance of research from aquatic and marine environments compared to terrestrial ecosystems, where relatively few evaluations exist, was highlighted by many scientists (Gnamuš et al. 2000; Bishop et al. 2020).

The knowledge of the Hg cycle and trophic transfer in terrestrial ecosystems in Poland, like in other regions of the world, is quite limited. Here, the focus is on THg accumulation in the terrestrial organisms from different trophic levels, especially those essential for the human diet. The selected elements include plants and macrofungi (Sect. Plants and fungi); freshwater fish (Sect. Freshwater fish); wild animals, including primary and secondary consumers (Sect. Wild animals); and livestock (Sect. Livestock) as the main source of animal protein for humans.

#### Plants and fungi

Terrestrial vegetation absorbs Hg primarily from the atmosphere via their stomata while the Hg uptake from the soil via roots is less significant (Stamenkovic and Gustin 2009). Terrestrial plants have been used as bioindicators of environmental Hg pollution for years. Mosses and lichens are particularly sensitive indicators, useful for determining the atmospheric deposition of Hg (Bargagli 2016; www.ivl.se), however, tree leaves are also commonly used for this purpose (De Nicola et al. 2013).

In wild-growing plants of Poland, the THg concentration was relatively low, which is confirmed, among others, by the studies on Hg in mosses. In unpolluted areas, both in the north and the south of Poland, the THg concentration in these plants ranged from 11 to 35 ng g^−1^ dw (Table S2a). These values were among the lowest values measured in mosses collected throughout Europe. Increased THg concentration was observed only in the mountains located in southeastern Poland, which is associated not only with the proximity of anthropogenic Hg sources (Fig. 2) but also with the increased natural Hg content in the bedrock (Pasieczna 2012). Higher THg concentrations were measured in the leaves of trees—Norway maple (*Acer plantanoides*) and large-leaved linden (*Tilia platyphyllos*), in which they ranged from 12 to 107 ng g^−1^ dw, amounting to 48 ng g^−1^ dw on average (Kowalski and Frankowski 2016). This is associated not only with the atmospheric Hg deposition but also with the transport of Hg to leaves via conductive tissues od trees. In the terrestrial plants, including grasses, herbs or tree leaves, the majority of Hg is present as a Hg(II), a slight proportion of MeHg is also detected in some cases. According to research conducted in the Czech Republic (Mališová et al. 2015) and Germany (Schwesig and Krebs 2003), the MeHg percentage in THg in the aboveground parts was lower (up to 0.5%) than in roots (up to 6%), which may be a result of the microbial methylation of Hg in the root-soil interface. Given the substantial biomass of trees, the litterfall from the deciduous plants at the end of the growing season to soils and the surface waters can be a significant source of Hg input to those systems. This load may increase due to longer exposure of leaves to atmospheric Hg during the year as the vegetation season in Poland becomes longer.

**Fig. 4:**
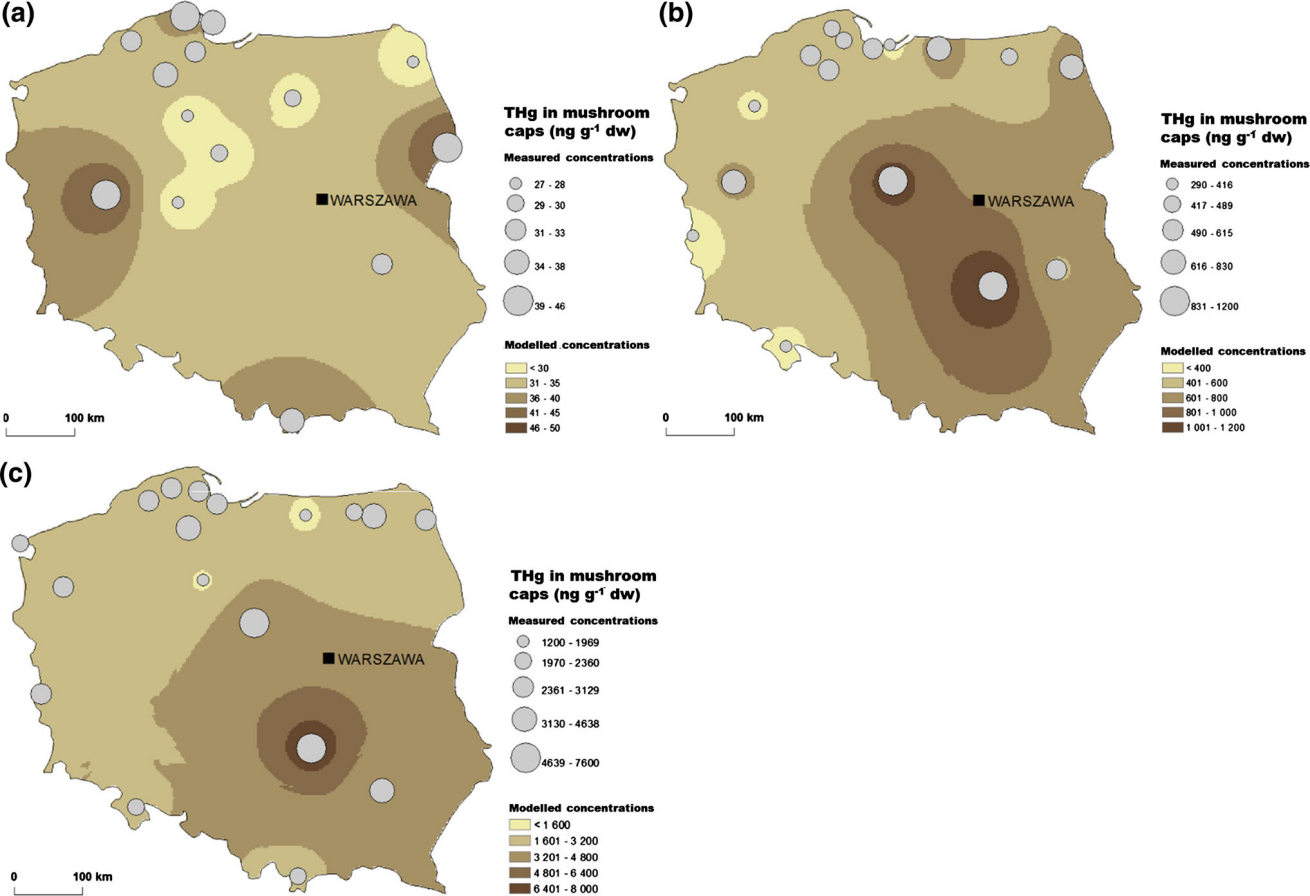
Total Hg (THg) concentration (ng g^−1^ dw) in caps of edible mushrooms from Poland in years 1994–2009: **a** common chanterelle, **b** brown birch bolete, and **c** king bolete (Falandysz and Chwir 1997; Falandysz and Bielawski 2007; Falandysz et al. 2003, 2007, 2012; Mazurkiewicz and Podlasińska 2014)

Macrofungi are important food for animals, including insects, snails, rodents, deer and wild boars and the fungal spores can account for up to 35% of their stomach volume (Nawrocka et al. 2020). Wild-growing mushrooms are also a popular component of the human diet, especially in Poland, which is also the second-largest producer of mushrooms in Europe. Among the popular edible species, the lowest mean THg concentration of 35 ng g^−1^ dw was measured in the common chanterelle (*Cantharellus* spp.) (Fig. 4a). In the caps of the brown birch bolete (*Leccinum scabrum*), it was twice as high and averaged 570 ng g^−1^ dw (Fig. 4b). The highest THg level, among investigated mushroom species, was measured in the king bolete (*Boletus edulis*) (Fig. 4c), in which the mean concentration was 3 000 ng g^−1^ dw, and in the parasol mushroom (*Macrolepiota procera*) with the mean THg concentration of 3 800 ng g^−1^ dw (Falandysz et al. 2007; Mazurkiewicz and Podlasińska 2014). These values are much higher than the safe level of THg for edible mushrooms, set by the Polish Ministry of Health, which amounts to 500 ng g^−1^ dw. The maximum THg concentrations measured in the caps of these fungi were more than double the mean value, which if consumed excessively, can lead to adverse health effects (Table S2c). Moreover, as shown by the study carried out in Switzerland, the contribution of MeHg in fruiting bodies of mushrooms can reach up to 11% of THg (Rieder et al. 2011). Given that the mushroom season in Europe is getting longer in recent decades, macrofungi can constitute available Hg source for animals throughout the whole year.

Numerous studies (Falandysz and Chwir 1997; Falandysz et al. 2007; Musilová et al. 2019) showed that mushrooms could act as bioindicators of Hg pollution. However, the process of Hg uptake by mushrooms is species-specific, and some taxa contain an elevated concentration of THg due to preferential bio-uptake from the soil. Based on the available literature data, it can be concluded that the brown birch bolete (*Leccinum scabrum*) can be used as an indicator species. The reason for this is the positive relationship between the THg concentration in the fungus tissue and the underlying substrate, especially in contaminated areas. In the case of this species, the increased levels of THg in caps correspond quite well to the increased THg level in soils and the location of point sources of Hg (Figs. 2, 4b).

#### Freshwater fish

**Fig. 5:**
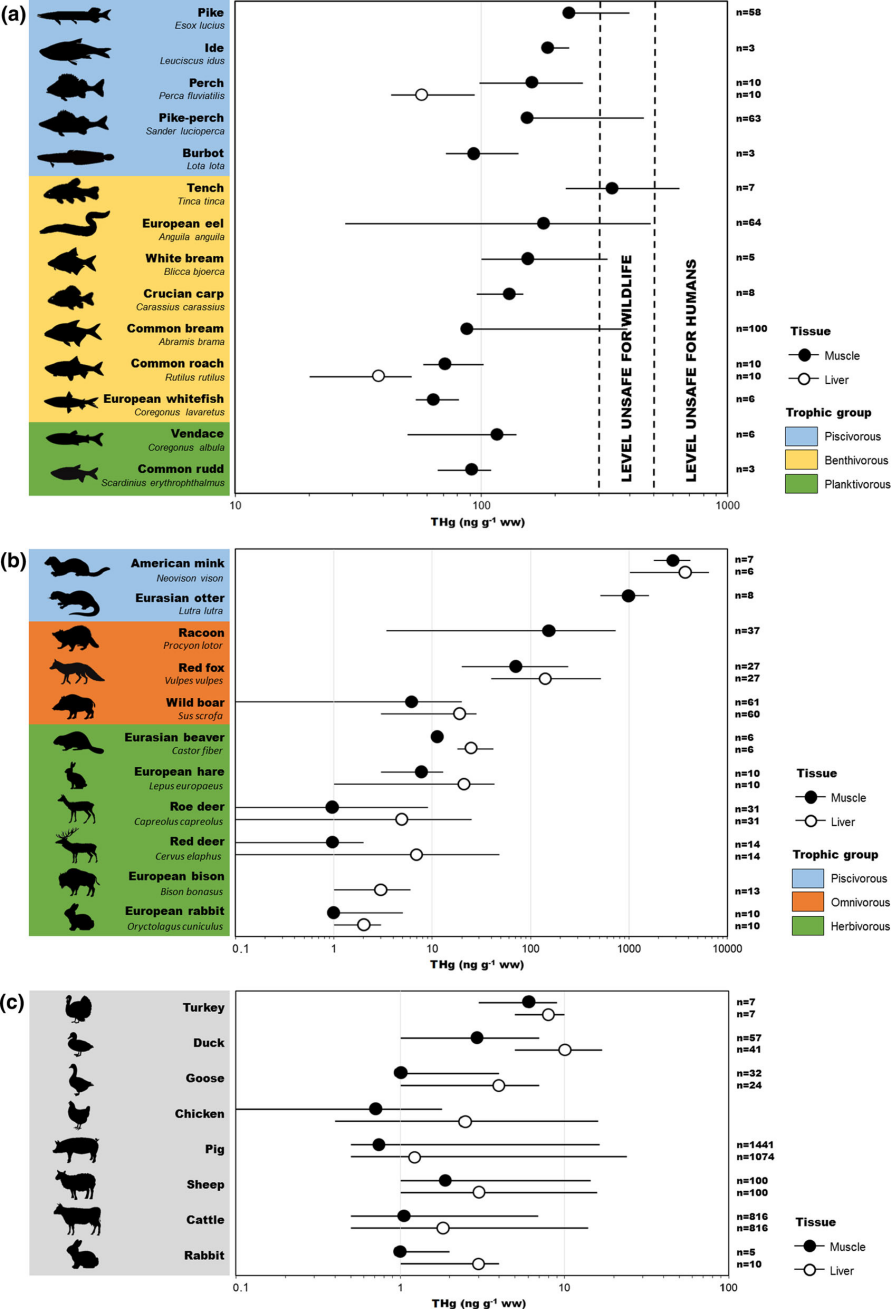
Total Hg (THg) concentration (ng g^−1^ ww) (mean and range) in muscles and livers of terrestrial animals with different feeding habits: **a** freshwater fish, **b** wild animals, and **c** livestock from Poland (values and references are given in the Table S4)

Research on freshwater fish in Poland showed that the trophic preferences of a given species had a great influence on THg concentration levels in fish muscles (Fig. 5a). Low THg concentration in planktivorous fish and high ones in predatory fish indicate the transfer of Hg in the aquatic food chain, a process which is emphasised by many authors who also point to the importance of species-specific characteristics (Lavoie et al. 2013; Wang et al. 2019). The highest THg level among predatory species was measured in muscles of pike (229 ng g^−1^ ww (wet weight)). It was similar in the case of Scandinavian lakes, although THg concentration in this species was 4 times higher than in Poland. In the case of other predatory species, the perch (*Perca fluviatilis*), THg concentration measured in Sweden and Finland was over twice as high as in Poland (Miller et al. 2013; Braaten et al. 2019). The possible explanation for the high THg concentration in fish from Scandinavia is elevated concentration of Hg in the underlying bedrock, as its geology strongly influences the Hg level in the water and hence in aquatic fauna (Danielsson et al. 2011; www.sgu.se). Elevated THg level in freshwater biota in Scandinavia may also by the result of forestry and other alterations of the landscape that promote the mobilisation of THg and MeHg and their transport from soils to surface waters and consequently greater bioaccumulation and biomagnification (Bishop et al. 2009; www.svo.se). THg concentration in predatory fish was close to the level found in benthivorous fish, such as the tench (*Tinca tinca*), the crucian carp (*Carassius carassius*) or the burbot (*Lota lota*) (Fig. 5a). Wyrzykowska et al. (2012) showed that in some species of benthic fish the THg level in muscles was similar to predatory fish and exceed the EU acceptable maximum levels of Hg in fish (500 ng g^−1^ ww). However, the mean THg concentration in the muscles of near-bottom freshwater fish in Poland was over 100 times lower than the concentration in fish of the Czech Republic and over 4 times greater than in Germany (Table S3a). Planktivorous fish caught in Poland, the common rudd (*Scardinius erythrophthalmus*) and the vendace (*Coregonus albula*), most often had a THg concentration in muscles below 100 ng g^−1^ ww (Fig. 5a). The results of the several years’ research indicate that the THg concentration in freshwater fish from Poland, in most cases were well below the permissible limit set out by the EU. According to the Water Framework Directive, the EQS concerning THg in fish should not exceed 20 ng g^−1^ ww. Unfortunately, in Poland, as in many other EU countries, the EQS for Hg are far from ‘good status’ (EEA 2018). Considering the risk to human health caused by freshwater fish consumption, but also the exposure of piscivorous animals, it is important to note that the majority of THg in fish tissues is MeHg. As shown by the studies conducted in the freshwater and brackish ecosystems of northern Poland, the percentage of MeHg in the muscle tissue of fish (i.e. perch, pike-perch, common roach, common bream) averaged 70–80% (Polak-Juszczak and Nermer 2016; Polak-Juszczak 2017). For comparison, in the muscles of fish collected from the polluted reservoir in the Czech Republic, almost 100% of THg was MeHg (Maršálek et al. 2005), which may be related to the usually occurring positive correlation between the THg concentration and the contribution of MeHg in the tissues and organs of fish. In the fish liver, the proportion of MeHg varied in a wide range from less than 30 to over 80%, which was connected to the efficiency of the demethylation in this organ (Polak-Juszczak 2017).

#### Wild animals

The available results for THg concentration in the tissues of wild terrestrial mammals representing different taxonomic units indicate a dependence on the trophic level. The lowest THg concentration (about 1 ng g^−1^ ww) was found in the muscle tissue of herbivores, including the European rabbit (*Oryctolagus cuniculus*), the roe deer (*Capreolus capreolus*) and the red deer (*Cervus elaphus*). Higher levels occurred in the muscle tissue of the European hare (*Lepus europaeus*) and the Eurasian beaver (*Castor fiber*) (Fig. 5b), which is probably because hares and beavers prefer foods with a higher Hg concentration—plants and crops rich in fat in the case of hares, and water macrophytes and tree bark in the case of beavers. Among omnivorous animals, elevated THg concentration was found in the muscle tissue of species for which predation is more dominant compared to more herbivorous species, such as the wild boar (*Sus scrofa*), the racoon (*Procyon lotor*) and the red fox (*Vulpes vulpes*) (Fig. 5b). Boars are highly versatile omnivores and the majority of their diet consists of food items dug from the ground, like plant material and burrowing animals. As omnivorous opportunistic feeders, raccoons feed on whatever is easiest to find, including carrion, rodents, molluscs and crustaceans, fish, injured waterfowl and their eggs and nestling. The diet of the red fox consists mainly of rodents, birds or ungulate carcasses. THg concentration in the tissues of primarily carnivorous mammals, including raccoon dogs (*Nyctereutes procyonoides*), badgers (*Meles meles*), pine martens (*Martes martes*), and polecat (*Mustela putorius*), varied from 40 to 230 ng g^−1^ ww in muscles and from 10 to 100 ng g^−1^ ww in livers (Kalisińska et al. 2009). In mammals closely related to aquatic environments, and feeding mainly on fish, such as the Eurasian otter (*Lutra lutra*) and the American mink (*Neovison vison*), the average THg concentration in muscle tissues was the highest and amounted to 972 and 2 801 ng g^−1^ ww, respectively (Kalisińska et al. 2012, 2017). As in the muscles, the average THg concentration in the livers of animals increased with trophic level from 2 ng g^−1^ ww in herbivorous rabbits to 3650 ng g^−1^ ww in piscivorous American minks (Fig. 5b). However, in the livers of mostly herbivorous and piscivorous animals, the THg concentration was higher than in muscles. The liver can accumulate Hg and plays an important role in its storage, redistribution, detoxification, and transformation. In mammals and birds, the liver is a major site for selenium induced Hg demethylation via the formation of HgSe (Lyytikäinen et al. 2015; Kalisińska et al. 2017). Moreover, the THg concentration in the tissues of piscivores were several orders of magnitude higher compared to herbivores. It reflects the Hg biomagnification occurring through diet, with a greater effect noticed in animals at higher trophic levels compared to those at lower trophic levels.

Herbivorous terrestrial mammals tend to have a very low contribution of MeHg in THg. As shown by research from Canada, in small grazers, like the hare, MeHg constituted about 3% of THg in muscles and 13% in livers. In the muscles of herbivore moose, the percentage of MeHg was slightly higher amounting to 13% (Golzadeh et al. 2020). In the case of roe deer from Slovenia, the percentage of MeHg was about 10% in muscles and 40% in livers (Gnamuš et al., 2020). Studies conducted in Northern America indicated that tissues of animals placed higher in the food chain, such as omnivorous ground squirrel and bear had a similar share of MeHg in THg to herbivores (Lasorsa and Allen-Gil 1995; Golzadeh et al. 2020). In the case of fish-eating mammals, such as mink and otter, the increase in the MeHg contribution to THg in their tissues and organs was significant and reached about 90% in the muscles and 60–80% in the livers (Evans et al. 2000; Strom 2008).

The impact of the pollution of the habitats of wild animals: the red deer, the roe deer and the wild boar on THg concentration in their muscles and livers was not significant (Table 1). Even in highly industrialised areas, such as the Upper Silesian Coal Basin (USCB), characterised by the presence of numerous hard coal mines and smelters (Fig. 2); the Bełchatów Industrial District (BID) with the largest lignite power plants in Europe and an open-cast mine; the Legnica-Głogów Copper District (LGCD), a copper mining and processing area, the THg concentration in animals was not much higher than in other regions (i.e. Falandysz 1994a; Szkoda et al. 2012; Durkalec et al. 2015, 2017, 2019). This is probably related to the Hg retention in the soil that protects the ecosystem from the full effect of the anthropogenic Hg atmospheric deposition (Bishop et al. 2009). As a result, the Hg transfer from soil into the food chain is limited. Concerning other areas of Europe, the THg concentration in the muscles of deer in Poland was higher than in Spain and Croatia (Table S3b). THg concentration in the muscles of wild boars from Poland was comparable to the THg level in the territory of Croatia and Lithuania, but it was almost three times lower than in Slovakia and central Spain (Table S3b). The current maximum allowable level of THg in meat derived from wild terrestrial vertebrates set by EU is 40 ng g^−1^ ww, except for wild boar liver, where the level is 100 ng g^−1^ ww. Given the available research results and the low annual intake of venison in Poland, the THg concentration found in the muscle and liver of wild animals should not pose a threat to consumer health.

**Table 1:** Total Hg (THg) concentration (ng g^−1^ ww) in muscle and liver of wild terrestrial animals from different regions of Poland: **a** red deer, **b** roe deer, and **c** wild boar (^1^Szkoda and Żmudzki 2001; ^2^Falandysz and Gajda 1988; ^3^Falandysz 1994a; ^4^Dobrowolska and Melosik 2002; ^5^Giżejewska et al. 2017; ^6^Albińska et al. 2011; ^7^Szkoda et al. 2012; ^8^Durkalec et al. 2015; ^9^Lech and Gubała 1998; ^10^Sobańska 2005)

		Region		Period	Muscle			Liver			Reference
					N	Mean	Range	N	Mean	Range	
A)	Red deer		Unknown	1998	74	2.0	< 10.0				1^*^
	*Cervus elaphus*			1999	82	3.0	< 32.0	218	6.0		1^*^
				2000	62	1.0	< 7.0				1^*^
		UP	Northern	1985–1986	76	2.0	1.0–24.0				2
				1987	102	1.1	0.9–10.0	28	8.3	2.2–17.0	3
				1988	87	1.9	0.4–7.7	32	9.1	2.0–35.0	3
				1989	78	0.8	0.3–2.2	10	3.5	2.0–5.9	3
				1990	76	0.9	0.3–3.3	10	2.7	2.0–3.3	3
				1991	60	1.3	0.3–3.5				3
			Northwestern	2000–2001				6	6.3	4.0–12.0	4
			Northeastern	2013–2014	14	1.0	0.1–2.0	14	7.0	0.1–48.0	5
		P	BID	2009	6	5.4	2.0–8.6	3	17.2	4.7–32.0	6
				2010	18	1.8	0.1–5.9	4	6.7	2.6–10.6	6
				2011–2012		0.1			3.0		7
			USCB	2011–2012		1.0			7.0		7
			LGCD	2011–2012		0.1			1.0		7
B)	Roe deer		unknown	1998	131	3.0	< 28.0				1^*^
	*Capreolus capreolus*			1999	140	2.0	< 30.0	398	7.0		1^*^
				2000	127	3.0	< 19.0				1^*^
		UP	Northern	1985–1986	102	2.0	1.0–13.0				2
				1987	149	1.3	0.4–10.0	28	10.0	2.0–36.0	3
				1988	84	2.8	0.6–18.0	45	10.0	1.7–65.0	3
				1989	99	1.2	0.3–4.5	10	6.5	2.3–11.0	3
				1990	89	1.3	0.3–2.5	10	11.0	1.7–23.0	3
				1991	59	1.1	0.4–3.5				3
			Northeastern	2011–2012		2.0			3.0		7
			Northeastern	2011–2013	31	1.0	0.1–9.0	31	5.0	0.1–25.0	8
			Southeastern	1996				14	3.3	0.0–11.7	9
		P	BID	2009	20	7.0	0.2–18.9	10	18.8	2.4–45.0	6
				2011–2012		1.0			8.0		7
			USCB	2011–2012		3.0			7.0		7
				2011–2013	26	1.0	0.1–6.0	25	8.0	1.0–33.0	8
			LGCD	2011–2012		4.0			16.0		7
				2011–2013	52	3.0	0.1–12.0	53	9.0	0.1–61.0	8
C)	Wild boar		Unknown	1998	186	7.0					1^*^
	*Sus scrofa*			1999	150	6.0		460	20.0		1^*^
				2000	124	7.0					1^*^
		UP	Northern	1985–1986	102	4.0	1.0–11.0	2	41.0	38.0–44.0	2
				1987	168	4.0	0.5–28.0	43	14.0	3.0–29.0	3
				1988	118	5.2	0.8–23.0	57	18.0	4.8–42.0	3
				1989	116	3.0	0.6–8.6	10	13.0	6.7–23.0	3
				1990	119	1.9	0.6–5.1	12	7.5	4.5–12.0	3
				1991	69	2.3	0.5–6.2				3
			Northeastern	1998–2000				17	9.0	5.0–22.0	10
				2011–2012		6.0			37.0		7
				2011–2013	61	6.0	0.1–20.0	60	19.0	3.0–28.0	8
				2000–2001				14	36.0	15.0–61.0	4
			Southeastern	1998–2000				8	20.0	14.0–25.0	10
		P	BID	2009	6	13.5	9.8–18.4				6
				2010	14	3.9	0.6–6.7	4	16.1	15.5–17.1	6
				2011–2012		5.0			16.0		7
			USCB	2011–2012		6.0			14.0		7
				2011–2013	51	7.0	0.1–35.0	52	27.0	0.1–231.0	8
			LGCD	1998–2000				15	4.2	1.0–6.0	10
				2011–2012		8.0			27.0		7
				2011–2013	56	7.0	0.1–23.0	56	26.0	6.0–84.0	8
			LSCB	1998–2000				20	21.0	4.0–16.0	10

*UP* unpolluted area, *P* polluted area, *USCB* Upper Silesian Coal Basin, *LSCB* Lower Silesian Coal Basin, *BID* Bełchatów Industrial District, *LGCD* Legnica-Głogów Copper District (to view locations, see Fig. 2)

^*^The minimum values were not given in this work

#### Livestock

Exposure of livestock to Hg depends on how they are kept and what breeding measures are used (EFSA 2008). An additional contribution, in particular for ruminants, may be constituted by the direct soil consumption, which can reach up to 40% of dry matter food intake (Smith et al. 2009). The average THg concentration in livestock muscles ranged from 1 to 6 ng g^−1^ ww depending on the species (Fig. 5c). The lowest THg level was found in the muscles of cattle, chickens, rabbits, geese and pigs. The THg concentration in the muscles of cattle in Poland was similar to those found in Spain, 5 times lower than in Sweden and 20 times lower compared to cattle from the Czech Republic (Table S3c). According to current regulations, the EU maximum level of THg permissible in the meat of livestock is 10 ng g^−1^ ww, whereas in offal it is 20 ng g^−1^ ww. The assessment of THg concentration in the muscle and liver of cattle and pigs carried out on behalf of the Polish government in 2009–2018 showed that only 0.4% of results were not acceptable. Therefore, the consumption of the meat of slaughter animals does not pose a significant threat to the health of consumers (Nawrocka et al. 2020). Probably due to the non-hazardous THg level in the Polish livestock, the Hg speciation was not examined. As shown by the study conducted in China, the share of MeHg in THg in poultry was low. For chicken, duck, and goose muscles it was up to 15, 25, and 40%, respectively. An even smaller proportion of MeHg was detected in livers of those birds, in which it did not exceed 20% THg (Yin et al. 2017). The poultry investigated in the aforementioned work was fed only with crops and vegetables, while in most European countries, including Poland, livestock feed is supplemented with fish meal. Therefore, meat from animals fed with fish meal or other fish products is likely to contain more MeHg (Lindberg et al. 2004).

### Temporal trends of biotic Hg

To estimate the time trends in biotic THg, this study presents only results of research from the period for which reliable data on Hg emission in Poland are available. Consequently, the paper refers to data published in the last three decades. For Poland, this period was not only a time of socio-political transformation, but also a time of changes in the industrial structure, the development of technology and science, and an increase in ecological awareness.

This section presents the temporal changes in Hg concentration in terrestrial plants (Sect. Terrestrial plants) based on the tree leaves results, and animals (Sect. Wild animals and livestock): game, including cervids and wild boar, and livestock, in particular cattle and pigs.

#### Terrestrial plants

**Fig. 6:**
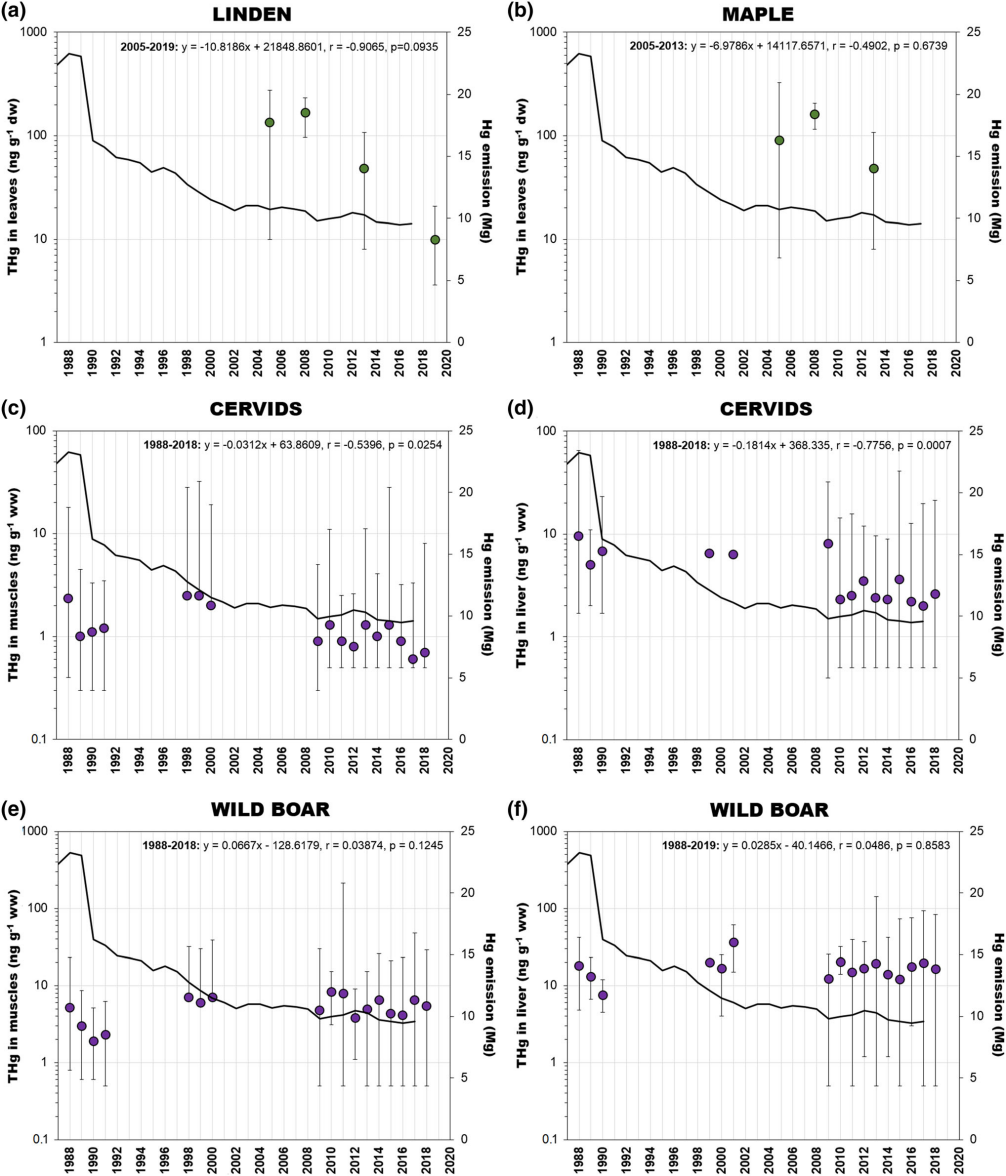
Temporal changes of total Hg (THg) concentration (mean and range) in terrestrial vegetation: **a** linden and **b** maple leaves (ng g^−1^ dw) muscles and livers of wild terrestrial animals: **c**, **d** cervids and **e**, **f** wild boar (ng g^−1^ ww) from Poland in relation to the anthropogenic emission of Hg (values and references are given in the Table S5). The data on the Hg national emission were taken from the KOBiZE (2019) inventory

The collected data do not provide sufficient information to discuss the long-term variability of THg concentration in terrestrial plants. The available data on the THg level in the leaves of linden (Fig. 6a) and maple (Fig. 6b) may indicate a downward trend. The mean THg concentration in linden leaves decreased from 134 ng g^−1^ in 2005 to 10 ng g^−1^ dw in 2019, which represents a decrease of 10 ng g^−1^ dw per year. In the case of maple leaves, the mean THg concentration dropped from 90 ng g^−1^ dw measured in 2005 to less than 50 ng g^−1^ dw in 2013 which gives a gradient of just under 7 ng g^−1^ dw year^−1^. THg concentration measured in linden leaves in 2005–2019 corresponded quite well with changes in Hg emissions (r = 0.87), which indicates their potential use as a bioindicator (Kowalski et al. 2012). Due to the small number of observations, the trends and correlations determined were statistically insignificant (*p* > 0.05).

The decrease in THg concentration in terrestrial vegetation may also be demonstrated by the values measured in moss, considered to be an environmental indicator. In the paper by Harmens et al. (2008), the THg concentration in samples from Poland in 1995 was very high and mostly exceeded 200 ng g^−1^ dw in almost every area of the country. Besides, the values presented for Poland were much higher than THg concentration measured in mosses collected in the same year in neighbouring countries. The results of research conducted in Poland in 2010–2012 indicate a decrease of more than tenfold in THg concentration in moss in unpolluted areas and a fourfold decrease in contaminated areas compared to 1995. The measured values were also lower than THg concentration in moss in most other European countries (Table S2).

#### Wild animals and livestock

Research conducted in the years 1988–2018 showed decreasing THg trends in muscle and liver of cervids (Fig. 6c, d). In these herbivorous animals, changes in THg concentration were correlated with the level of Hg emission (Table 2). The significant decrease in Hg emissions that occurred in the last decade of the previous century (Fig. 1) probably caused a decrease in the Hg concentration in plants (Fig. 6a, b) reducing the exposure of herbivorous red deer and roe deer. This is also indicated by statistically significant differences in THg concentration in cervids between periods with different Hg emission (Fig. S2), both for muscle and liver (Table 3). For both tissues, Hg concentrations during the period of stabilised Hg emission were about 2 times lower than before. Contrary to cervids, a negative relationship between Hg emission and THg concentration was found in the muscles of wild boar (Table 2; Fig. 6e). In the case of wild boar liver, the THg concentrations were not dependent on time or Hg emission (Table 2; Fig. 6f). For both tissues, differences in Hg concentration during periods with different Hg emission, were not statistically significant (Table 3). To indicate possible causes affecting strong fluctuations in THg concentration in wild boar in recent years, more samples from different regions of Poland and differences in age of animals in individual years should be presented. Another possible explanation may be the large diversity of food consumed by wild boar in their living areas. The diet of wild boar consists mainly of plants, but also the food of animal origin, among others carrion, invertebrates, birds and small mammals. Moreover, their diet changes seasonally which differentiates the food exposure to Hg through the year. The rooting by wild boar' is also a factor that can both increase and decrease the Hg load entering the animals’ body with the soil they ingest from different areas (Fig. 2). The phenomenon of geophagia, defined as consumption of earth or soil-like substances, has been described on the example of ruminants for which soil can account for up to 40% of dry matter content (Smith et al. 2009). The THg concentrations in wild boar tissues may also have been influenced by processes that are not dose-dependent and unrelated to the food intake, such as Hg detoxification and elimination (Bełdowska and Falkowska 2016).

**Table 2:** Relationship between total Hg (THg) concentration in terrestrial organisms from Poland and the national Hg emission

		Period	Equation	r	p
Wild Animals	Wild boar—muscles	1988–2018	Hg_biota_ = 0.1625Hg_emission_ + 7.3135	0.4709	0.0418
	Wild boar—liver	1988–2018	Hg_biota_ = 0.2193Hg_emission_ + 15.3465	0.1454	0.5650
	Cervids—muscles	1988–2018	Hg_biota_ = 0.0478Hg_emission_ + 0.5577	0.5360	0.0266
	Cervids—liver	1988–2018	Hg_biota_ = 0.3595Hg_emission_ + 0.0444	0.6967	0.0027
Livestock	Cattle—muscles	1988–2018	Hg_biota_ = 0.0549Hg_emission_ + 0.232	0.7164	0.0039
	Cattle—liver	1988–2018	Hg_biota_ = 0.2101Hg_emission_− 0.0924	0.8184	0.0001
	Pig—muscles	1988–2018	Hg_biota_ = 0.1257Hg_emission_− 0.5323	0.8476	0.0000
	Pig—liver	1988–2018	Hg_biota_ = 0.2457Hg_emission_− 1.2032	0.9528	0.0000

**Table 3:** Results of Mann–Whitney Test and the statistical comparison of total Hg (THg) concentration in muscle and liver of wild terrestrial animals and livestock in period of stabilised Hg emission and before. These periods were distinguished on the basis of the data presented in Fig. 1 and Fig. S2

		Mean THg (ng g^−1^ ww)		
		Until 2002	After 2002	*p*
Wild animals	Wild boar—muscles	4.6	5.7	0.4642
	Wild boar—liver	18.5	16.3	0.7449
	Cervids—muscles	1.8	1.0	0.0147*
	Cervids—liver	6.8	3.2	0.0120*
Livestock	Cattle—muscles	1.3	1.1	0.2888
	Cattle—liver	4.6	1.8	0.0058*
	Pig—muscles	1.8	0.7	0.0072*
	Pig—liver	3.5	1.2	0.0058*

^*^The difference between two period is statistically significant at the p-value < 0.05

**Fig. 7:**
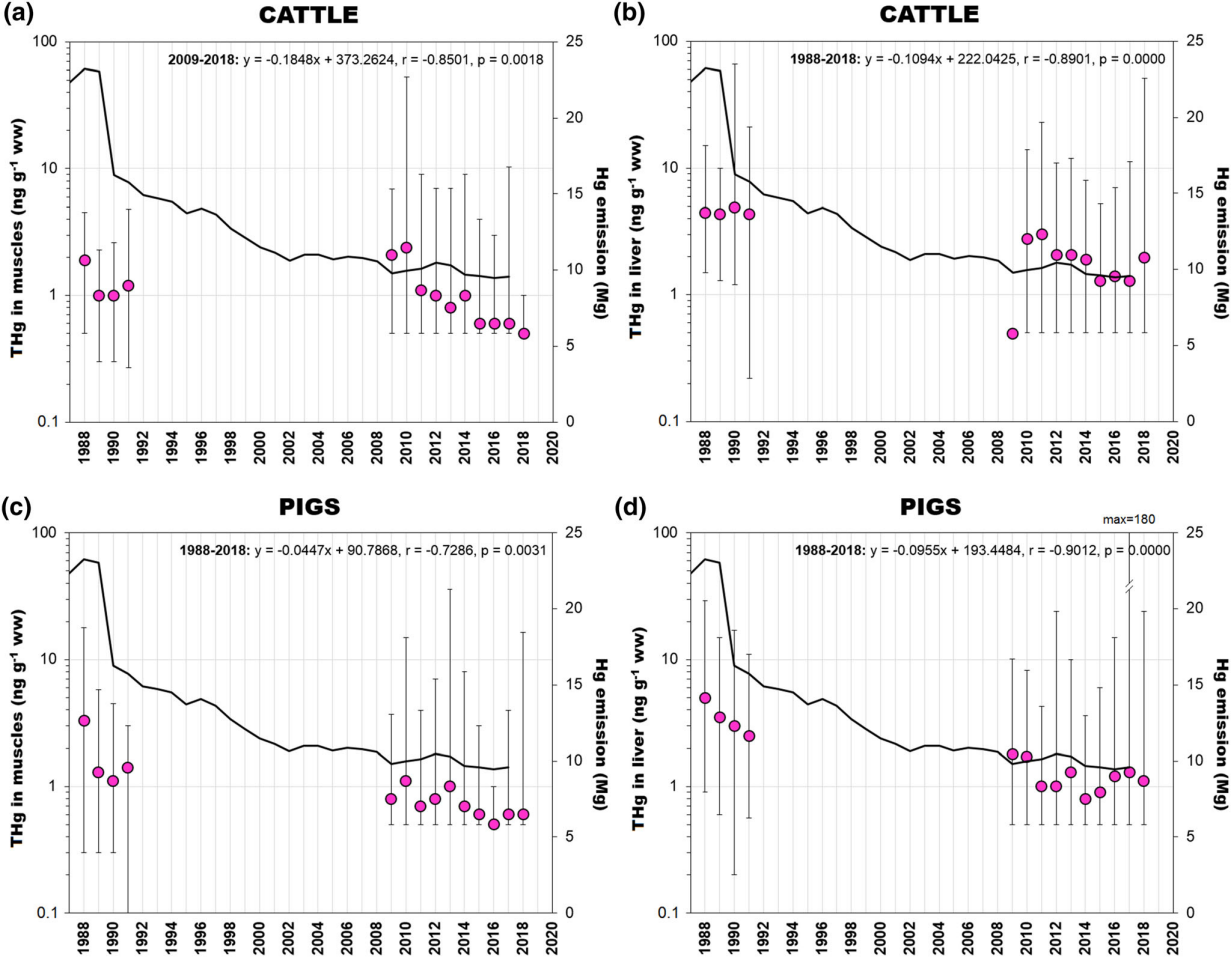
Temporal changes of total Hg (THg) concentration (mean and range) in muscles and livers of livestock: **a**, **b** cattle, and **c**, **d** pigs (ng g^−1^ ww) from Poland in relation to the anthropogenic emission of Hg (values and references are given in the Table S5). The data on the Hg national emission were taken from the KOBiZE (2019) inventory

The meat composition of livestock has been investigated in Poland since the mid-1970s. The obtained results indicate that THg concentration in tissues of cattle and pigs in the last three decades are getting lower (Fig. 7a, b, c, d). Differences in THg concentration during and before the stabilisation of Hg emission were statistically significant for cattle livers, and pig muscles and livers (Table 3). In each of these tissues, the average THg concentration decreased by more than twofolds. The observed drop in THg concentration in cattle and pig tissues reflects a reduction in Hg emission, which is confirmed by a significant correlation between THg concentration in both the liver and muscle of livestock and the level of emission (Table 2). At the same time, the food exposure of farm animals to Hg was reduced due to the elimination of plant protection products based on Hg compounds, which were used for the seeds conservation and field spraying. These substances were withdrawn from the EU market in the late 1970s, however, in Poland Hg was used widely for seed preservation until the 1980s (Falandysz 1994b). The current level of Hg in cultivated plants may result from the use of some fertilisers or soil amendments (Sánchez-Báscones et al. 2017). In contrast, food additives, fish-based proteins and others, such as protein hydrolysates from poultry and protein made from feathers may constitute an additional source of organic Hg. Inorganic Hg, by contrast, may be present in mineral supplements, but they usually occur in amounts not exceeding the maximum allowable levels of this element (Hedegaard and Sloth 2011). The reduction of THg concentration in tissues of livestock over the years could also be influenced by the EU ban on the use of meat and bone meal (MBM) of mammals in animal feeds. The regulation was introduced in 2001 and applied to all livestock, including fish (EC 2001). The THg level in MBM is generally much higher compared to other animal feeding stuff, especially plant-based ones such as soybean or maize (EFSA 2008).

Among the factors that could significantly contribute to a decrease in THg concentration in livestock, in addition to reduced Hg emission, one should mention the development of breeding technology and the increase of requirements in the control of contamination levels in fodder and feed materials together with the implementation of analytical methods enabling reduction of costs and examination time. Despite the indication of a downward trend in the THg level in livestock, the Hg concentration in the food of animal origin should be constantly monitored to ensure an adequate level of consumer safety.

## Conclusion

As indicated in HELCOM (2018) and EMEP (2018) reports, Poland is one of the main emitters of Hg to the atmosphere among the Baltic Sea region countries. It is also the country where the reduction of Hg emission is least effective compared to most of Europe (EEA 2019), which proves the urgent need for modernisation and transformations in the energy and industry sectors. However, analyses of organisms inhabiting both the terrestrial and aquatic environment indicated a moderate or low THg concentration level compared to other places on the globe. This inconsistency may be caused by the insufficient implementation of the atmospheric Hg monitoring programme in Poland and transfer of these data into global databases. The uncertainty of the international model calculations of atmospheric deposition may result from the lack of accurate data from Polish atmospheric Hg monitoring. Besides the European Monitoring and Evaluation Programme (EMEP), Poland also participates in the Global Atmosphere Watch Programme (GAW) of the World Meteorological Organization and the COMBINE Programme of HELCOM, which provide information on the environmental quality to the public and to policy-makers. As a part of the Polish State Environmental Monitoring Programme, measurements of TGM and THg in precipitation are carried out at five background stations, which are evenly distributed throughout the country. However, only one of these stations, located in the northeastern part of the country, is in compliance with the EMEP requirements. Therefore, the amount of valuable data from Polish monitoring included in the international network is insufficient, making model results verification very difficult. The need for validation and calibration of the models used in the most sensitive areas is notable. The another reason of low THg concentration in terrestrial organisms is probably connected with relatively low bioavailability of Hg in abiotic compartments. It is likely that Hg deposited on Polish territory is retained in a less mobile form, mainly bound in organic-rich soils. Environmental conditions in Poland indicate that in soils, its dominant form is inorganic Hg, which is only slightly transformed into MeHg. Consequently, this limits the Hg uptake by terrestrial biota and makes the organic-rich topsoil the main Hg storage in Poland. However, considering the direction of environmental changes, related mainly to the climate, it is forecast that as a result of remobilisation, the Hg outflow from the soil to rivers and consequently into the sea may intensify in the next few years. It is crucial for inland and coastal waters, for which rivers constitute a major source of Hg.

The research and monitoring of current status and trends of THg contamination in terrestrial organisms in Poland are still insufficient, especially when compared with countries such as Sweden or Canada, where regular monitoring of Hg pollution, including the use of bioindicators, began already in the 1960s (www.ivl.se). The authors selected organisms that can be bioindicators of THg pollution and be useful for identification of Hg ‘hot-spots’, namely brown birch bolete mushroom (*Luccinum scabrum*) and herbivorous wild animals, mainly deer (Cervidae). Although the recent THg level in fish and wild animals from Poland poses no risk to consumers, systematic and thoughtful monitoring is necessary, as it would allow the prediction of the consequences of climate change on the Hg cycle in the environment and the effects of this process on humans. This fully complies with the provisions of the Minamata Convention.

## Supplementary Information

Below is the link to the electronic supplementary material.

Supplementary file1 (PDF 956 KB)

## References

[CR1] Albińska J, Góralski J, Szynkowska MI, Leśniewska E, Paryjczak T (2011). Mercury in carcasses of wild animals hunted in the province of Lodz. Rocznik Ochrona Środowiska.

[CR2] Baralkiewicz D, Gramowska H, Godyn R (2006). Distribution of total mercury and methylmercury in water, sediment and fish from Swarzdzkie lake. Chemistry and Ecology.

[CR3] Bargagli R (2016). Moss and lichen biomonitoring of atmospheric mercury: A review. Science of The Total Environment.

[CR4] Bełdowska M, Falkowska L (2016). Mercury in marine fish, mammals, seabirds, and human hair in the coastal zone of the southern Baltic. Water, Air, and Soil Pollution.

[CR5] Bełdowska M, Jędruch A, Łęczyński L, Saniewska D, Kwasigroch U (2016). Coastal erosion as a source of mercury into the marine environment along the Polish Baltic shore. Environmental Science and Pollution Research.

[CR6] Bishop K, Allan CJ, Bringmark L, Garcia E, Hellsten S, Högbom L, Johansson L, Lomander A, Nordin Y (2009). Forestry’s contribution to Hg bioaccumulation in freshwaters: assessment of the available evidence. Does forestry contribute to mercury in Swedish fish?.

[CR7] Bishop K, Shanley JB, Riscassi A, de Wit HA, Eklöf K, Meng B, Mitchell C, Osterwalder S (2020). Recent advances in understanding and measurement of mercury in the environment: Terrestrial Hg cycling. Science of The Total Environment.

[CR8] Braaten HFV, de Wit HA, Fjeld E, Rognerud S, Lydersen E, Larssen T (2014). Environmental factors influencing mercury speciation in Subarctic and Boreal lakes. Science of The Total Environment.

[CR9] Braaten HFV, Åkerblom S, Kahilainen KK, Rask M, Vuorenmaa J, J., Mannio, T. Malinen, E. Lydersen, (2019). Improved Environmental Status: 50 Years of Declining Fish Mercury Levels in Boreal and Subarctic Fennoscandia. Environmental Science and Technology.

[CR10] Bravo AG, Kothawala DN, Attermeyer K, Tessier E, Bodmar P, Lefesma JLJ, Audet J, Casas-Ruiz JP (2018). The interplay between total mercury, methylmercury and dissolved organic matter in fluvial systems: A latitudinal study across Europe. Water Research.

[CR11] Danielsson, S., J. Hedman, A. Miller, and A. Bignert. 2011. Mercury in Perch from Norway, Sweden and Finland – Geographical Patterns and Temporal Trends. Swedish Museum of Natural History, Report 8/2011, Stockholm, Sweden.

[CR12] De Nicola F, Spagnuolo V, Baldantoni D, Sessa L, Alfani A, Bargagli R, Monaci F, Terracciano S (2013). Improved biomonitoring of airborne contaminants by combined use of holm oak leaves and epiphytic moss. Chemosphere.

[CR14] Dobrowolska A, Melosik M (2002). Mercury contents in liver and kidneys of wild boar (*Sus scrofa*) and red deer (*Cervus elaphus*). Zeitschrift für Jagdwissenschaft.

[CR15] Driscoll CT, Holsapple J, Schofield CL, Munson R (1998). The chemistry and transport of mercury in a small wetland in the Adirondack region of New York, USA. Biogeochemistry.

[CR16] Durkalec M, Szkoda J, Kolacz R, Opalinski S, Nawrocka A, Żmudzki J (2015). Bioaccumulation of Lead, Cadmium and Mercury in Roe Deer and Wild Boars from Areas with Different Levels of Toxic Metal Pollution. International Journal of Environmental Research.

[CR17] Durkalec M, Kolenda R, Owczarek T, Szkoda J, Nawrocka A, Grzegrzółka J, Dzięgiel P, Socha P (2017). Expression of metallothionein in the liver and kidneys of the red deer (*Cervus elaphus* L.) from an industrial metal smelting area of Poland. Ecotoxicology and Environmental Safety.

[CR18] Durkalec M, Nawrocka A, Żmudzki J, Filipek A, Niemcewicz M, Posyniak A (2019). Concentration of Mercury in the Livers of Small Terrestrial Rodents from Rural Areas in Poland. Molecules.

[CR19] EC. 2001. Regulation 999/2001 of the European Parliament and of the Council of 22 May 2001 laying down rules for the prevention, control and eradication of certain transmissible spongiform encephalopathies. *OJ L* 147: 1–40. http://data.europa.eu/eli/reg/2001/999/oj

[CR13] EC, 2008. Directive 2008/105 of the European Parliament and of the Council of 16 December 2008 on environmental quality standards in the field of water policy. *OJ L* 348: 84–97. http://data.europa.eu/eli/dir/2008/105/oj

[CR20] EC. 2017. Regulation 2017/852 of the European Parliament and of the Council of 17 May 2017 on mercury. *OJ L* 137: 1–21. http://data.europa.eu/eli/reg/2017/852/oj

[CR21] EEA (2018). European waters. Assessment of status and pressures.

[CR22] EEA. 2019. Data and maps catalogue. Retrieved 25 September, 2020, from https://www.eea.europa.eu/data-and-maps/daviz/change-in-emissions-of-heavy-metals

[CR23] EFSA (2008). Mercury as undesirable substance in animal feed - Scientific opinion of the Panel on Contaminants in the Food Chain. EFSA Journal.

[CR24] Eklöf K, Fölster J, Sonesten L, Bishop K (2012). Spatial and temporal variation of THg concentrations in run-off water from 19 boreal catchments, 2000–2010. Environmental Pollution.

[CR25] EMEP. 2016. Atmospheric Supply of Nitrogen, Cadmium, Mercury, Benzo(a)pyrene and PBDEs to the Baltic Sea in 2014. Technical Report MSC-W 1/2016, Oslo, Norway.

[CR26] EMEP. 2018. Atmospheric Supply of Nitrogen, Cadmium, Mercury, Benzo(a)pyrene, and PCB-153 to the Baltic Sea in 2016. Technical Report MSC-W 1/2018, Oslo, Norway.

[CR27] Emmerton CA, Cooke CA, Wentworth GR, Graydon JA, Ryjkov A, Dastoor A (2018). Total Mercury and Methylmercury in Lake Water of Canada’s Oil Sands Region. Environmental Science and Technology.

[CR29] Evans RD, E.M., Addison, J.Y. Villeneuve, K.S. MacDonald, and D.G. Joachim. (2000). Distribution of Inorganic and Methylmercury among Tissues in Mink (*Mustela vison*) and Otter (*Lutra canadensis*). Environmental Research.

[CR30] Falandysz J (1994). Some toxic and trace metals in big game hunted in the northern part of Poland in 1987–1991. Science of The Total Environment.

[CR31] Falandysz J, Renzoni A, Mattei N, Lari L, Fossi C (1994). The Uses of Pesticides and Their Levels in Food in Eastern Europe: The Example of Poland. Contaminants in the Environment: A Multidisciplinary Assessment of Risks to Man and Other Organisms.

[CR32] Falandysz J, Bielawski L (2007). Mercury and its bioconcentration factors in Brown Birch Scaber Stalk (*Leccinum scabrum*) from various sites in Poland. Food Chemistry.

[CR33] Falandysz J, Chwir A (1997). The concentrations and bioconcentration factors of mercury in mushrooms from the Mierzeja Wiślana sand-bar, Northern Poland. Science of The Total Environment.

[CR34] Falandysz J, Gajda B (1988). Mercury content in muscle, liver and kidneys of slaughtered and game animals from the northern part of Poland, 1985–1986. Roczniki PZH.

[CR35] Falandysz J, Kawano M, Świeczkowski A, Brzostowski A, Dadej M (2003). Total mercury in wild-grown higher mushrooms and underlying soil from Wdzydze Landscape Park, Northern Poland. Food Chemistry.

[CR36] Falandysz J, Frankowska A, Mazur A (2007). Mercury and its bioconcentration factors in King Bolete (*Boletus edulis*) Bull Fr. Journal of Environmental Science and Health, Part A.

[CR108] Falandysz, J., E. Widzicka, A.K. Kojta, G. Jarzyńska, M. Drewnowska, A. Dryżałowska, D. Danisiewicz-Czupryńska, E. Lenz, et al. 2012. Mercury in Common Chanterelles mushrooms: *Cantharellus* spp. update. *Food Chemistry* 133: 842–850. 10.1016/j.foodchem.2012.01.102.

[CR37] FOREGS (2005). Geochemical Atlas of Europe Part 1–Background Information, Methodology and Maps.

[CR38] Gajewska RE, Nabrzyski M (1977). Mercury, cadmium and lead content in sea and freshwater fish. Roczniki PZH.

[CR39] Gębka K, Bełdowska M, Saniewska D, Kuliński K, Bełdowski J (2018). Watershed characteristics and climate factors effect on the temporal variability of mercury in the southern Baltic Sea rivers. Journal of Environmental Sciences.

[CR40] Gębka K, Saniewska D, Bełdowska M (2020). Mobility of mercury in soil and its transport into the sea. Environmental Science and Pollution Research.

[CR41] Giżejewska A, Szkoda J, Nawrocka A, Żmudzki J, Giżejewski Z (2017). Can red deer antlers be used as an indicator of environmental and edible tissues’ trace element contamination?. Environmental Science and Pollution Research.

[CR42] Gnamuš A, Bryne AR, Horvat M (2000). Mercury in the Soil-Plant-Deer-Predator Food Chain of a Temperate Forest in Slovenia. Environmental Science and Technology.

[CR43] Golzadeh N, Brast BD, Basu N, Baker JM, Auger JC (2020). Evaluating the concentrations of total mercury, methylmercury, selenium, and selenium:mercury molar ratios in traditional foods of the Bigstone Cree in Alberta Canada. Chemosphere.

[CR44] Harmens H, Norris DA, Koerber GR, Buse A, Steinnes E, Rühling Å (2008). Temporal trends (1990–2000) in the concentration of cadmium, lead and mercury in mosses across Europe. Environmental Pollution.

[CR45] Hedegaard RV, Sloth JJ (2011). Speciation of arsenic and mercury in feed: Why and how?. Biotechnology, Agronomy, Society and Environment.

[CR46] HELCOM (2018). Inputs of hazardous substances to the Baltic Sea.

[CR47] Hławiczka S (2008). Rtęć w środowisku atmosferycznym.

[CR48] Holmes CD, Jacob DJ, Yang X (2006). Global lifetime of elemental mercury against oxidation by atomic bromine in the free troposphere. Geophysical Research Letters.

[CR49] Jędruch A, Kwasigroch U, Bełdowska M, Kuliński K (2017). Mercury in suspended matter of the Gulf of Gdańsk: Origin, distribution and transport at the land–sea interface. Marine Pollution Bulletin.

[CR50] Jędruch A, Bełdowska M, Ziółkowska M (2019). The role of benthic macrofauna in the trophic transfer of mercury in a low-diversity temperate coastal ecosystem (Puck Lagoon, southern Baltic Sea). Environmental Monitoring and Assessment.

[CR51] Kalisińska E, Lisowski P, Salicki W, Kucharska T, Kavetska K (2009). Mercury in wild terrestrial carnivorous mammals from north-western Poland and unusual fish diet of red fox. Acta Theriologica.

[CR52] Kalisińska E, Budis H, Lanocha N, Podlasinska J, Jedrzejewska E, Kosik-Bogacka DI (2012). Comparison of Hepatic and Nephric Total Mercury Concentrations Between Feral and Ranch American Mink (*Neovison vison*) from Northwestern Poland. Bulletin of Environmental Contamination and Toxicology.

[CR53] Kalisińska E, Lanocha-Arendarczyk N, Kosik-Bogacka DI, Budis H, Pilarczyk B, Tomza-Marciniak A, Podlasinska J (2017). Muscle mercury and selenium in fishes and semiaquatic mammals from a selenium-deficient area. Ecotoxicology and Environmental Safety.

[CR54] KOBiZE. (2019). Poland’s Informative Inventory.

[CR55] Korejwo E, Saniewska D, Bełdowska M (2020). Fractionation of mercury in aerosols of the southern Baltic coastal zone. Atmospheric Environment.

[CR56] Kossakowski S (1979). Dynamics of the distribution of mercury in the animal organisms. Polskie Archiwum Weterynaryjne.

[CR57] Kowalski A, Frankowski M (2016). Seasonal variability of mercury concentration in soils, buds and leaves of *Acer platanoides* and *Tilia platyphyllos* in central Poland. Environmental Science and Pollution Research.

[CR58] Kowalski A, Siepak M, Boszke L (2007). Mercury contamination of surface and ground waters of Poznan, Poland. Polish Journal of Environmental Studies.

[CR59] Kowalski A, Frankowski M, Zioła-Frankowska A, Mocek-Płóciniak A, Siepak J (2012). Variability of mercury concentrations in soil and leaves of *Acer plantanoides* and *Tilia platyphyllos* in Poznań city, Poland. Soil and Sediment Contamination.

[CR60] Larssen T, de Wit HA, Wiker M, Halse K (2008). Mercury budget of a small forested boreal catchment in southeast Norway. Science of The Total Environment.

[CR61] Lasorsa B, Allen-Gil S (1995). The Methylmercury to Total Mercury Ratio In Selected Marine, Freshwater, and Terrestrial Organisms. Water, Air, and Soil Pollution.

[CR62] Lavoie RA, Jardine TD, Chumchal MM, Kidd KA, Campbell LM (2013). Biomagnification of mercury in aquatic food webs: a worldwide meta-analysis. Environmental Science and Technology.

[CR63] Lech T, Gubała W (1998). Metale ciężkie w wątrobie i nerkach saren z regionu województwa krakowskiego. Bromatologia i Chemia Toksykologiczna.

[CR64] Lindberg A, Björnberg KA, Vahter M, Berglund M (2004). Exposure to methylmercury in non-fish-eating people in Sweden. Environmental Research.

[CR65] Lindström M (2001). Distribution of particulate and reactive mercury in surface waters of Swedish forest lakes - an empirically based predictive model. Ecological Modelling.

[CR66] Lyytikäinen M, Pätynen J, Hyvärinen H, Sipilä T, Kunnasranta M (2015). Mercury and Selenium Balance in Endangered Saimaa Ringed Seal Depend on Age and Sex. Environmental Science and Technology.

[CR67] Mališová K, Koplík R, Mestek O (2015). Speciation of Mercury in Terrestrial Plants Using Vapor Generation and Liquid Chromatography-Inductively Coupled Plasma Mass Spectrometry. Analytical letters.

[CR68] Maršálek P, Svobodová Z, Randák T, Švehla J (2005). Mercury and Methylmercury Contamination of Fish from the Skalka Reservoir: A Case Study. Acta Veterinaria Brno.

[CR69] Mazurkiewicz N, Podlasińska J (2014). The mercury content of macrofungi from area of west Pomeranian district. Bromatologia i Chemia Toksykologiczna.

[CR70] Miller A, Bignert A, Porvari P, Danielsson S, Verta M (2013). Mercury in Perch (*Perca fluviatilis*) from Sweden and Finland. Water, Air, and Soil Pollution.

[CR71] Musilová J, Trebichalský P, Jančo I, Tóth T, Šnirc M (2019). Mercury bioaccumulation in *Boletus edulis* bull. in different forest ecosystems in Slovakia. GeoConference SGEM.

[CR72] Nabrzyski M (1975). Mercury, copper, and zinc content in the meat tissue of some freshwater fish. Bromatologia i Chemia Toksykologiczna.

[CR73] Nawrocka A, Durkalec M, Szkoda J, Filipek A, Kmiecik M, Żmudzki J, Posyniak A (2020). Total mercury levels in the muscle and liver of livestock and game animals in Poland, 2009–2018. Chemosphere.

[CR74] Pacyna EG, Pacyna JM, Sundseth K, Munthe J, Kindbom K, Wilson S, Steenhuisen F, Maxson P (2010). Global emission of mercury to the atmosphere from anthropogenic sources in 2005 and projections to 2020. Atmospheric Environment.

[CR75] Pasieczna A (2012). Polish geochemical atlas.

[CR76] Petersen G, Linkov I, Schell WR (1999). Airborne Heavy Metals over Europe: Emissions, Long-Range Transport and Deposition Fluxes to Natural Ecosystems. Contaminated Forests.

[CR77] Pirrone N, Keeler GJ, Nriagu JO (1996). Regional differences in worldwide emissions of mercury to the atmosphere. Atmospheric Environment.

[CR78] Pirrone N, Cinnirella S, Feng X, Finkelman RB, Friedli HR, Leaner J, Mason R, Mukherjee AB (2010). Global mercury emissions to the atmosphere from anthropogenic and natural sources. Atmospheric Chemistry and Physics.

[CR79] Pogrzeba M, Ciszek D, Galimska-Stypa R, Nowak B, Sas-Nowosielska A (2016). Ecological strategy for soil contaminated with mercury. Plant and Soil.

[CR80] Polak-Juszczak L (2017). Methylmercury in fish from the southern Baltic Sea and coastal lagoons as a function of species, size, and region. Toxicology and Industrial Health.

[CR81] Polak-Juszczak L, Nermer T (2016). Methylmercury and Total Mercury in Eels, *Anguilla anguilla*, from Lakes in Northeastern Poland: Health Risk Assessment. EcoHealth.

[CR82] Pyta H, Widziewicz-Rzońca K, Słaby K (2020). Inhalation exposure to gaseous and particulate bound mercury present in the ambient air over the polluted area of southern Poland. International Journal of Environmental Research and Public Health.

[CR83] Rafaj P, Bertok I, Cofala J, Schöpp W (2013). Scenarios of global mercury emissions from anthropogenic sources. Atmospheric Environment.

[CR84] Rieder SR, Brunner I, Horvat M, Jacobs A, Frey B (2011). Accumulation of mercury and methylmercury by mushrooms and earthworms from forest soils. Environmental Pollution.

[CR85] Saba M, Falandysz J, Nnorom IC (2016). Mercury bioaccumulation by *Suillus bovinus* mushroom and probable dietary intake with the mushroom meal. Environmental Science and Pollution Research.

[CR86] Sánchez-Báscones M, Antolín-Rodríguez JM, Martín-Ramos P, González-González A, Bravo-Sánchez CT, Martín-Gil J (2017). Evolution of mercury content in agricultural soils due to the application of organic and mineral fertilizers. Journal of Soils and Sediments.

[CR87] Saniewska D, Bełdowska M, Bełdowski J, Falkowska L (2014). Mercury in precipitation at an urbanised coastal zone of the Baltic Sea (Poland). Ambio.

[CR88] Saniewska D, Bełdowska M, Bełdowski J, Saniewski M, Szubska M, Romanowski A, Falkowska L (2014). The impact of land use and season on the riverine transport of mercury into the marine coastal zone. Environmental Monitoring and Assessment.

[CR89] Saniewska D, Bełdowska M, Bełdowski J, Jędruch A, Saniewski M, Falkowska L (2014). Mercury loads into the sea associated with extreme flood. Environmental Pollution.

[CR90] Saniewska D, Bełdowska M, Bełdowski J, Saniewski M, Gębka K, Szubska M, Wochna A (2018). Impact of intense rains and flooding on mercury riverine input to the coastal zone. Marine Pollution Bulletin.

[CR91] Saniewska D, Gębka K, Bełdowska M, Siedlewicz G, Bełdowski J (2019). Impact of hydrotechnical works on outflow of mercury from the riparian zone to a river and input to the sea. Marine Pollution Bulletin.

[CR92] Schwesig D, Krebs O (2003). The role of ground vegetation in the uptake of mercury and methylmercury in a forest ecosystem. Plant and Soil.

[CR93] Skotak K, Degórska A, Prządka Z, Syrzycki M (2019). Assessment of air pollution with mercury at regional background stations in Poland for 2018.

[CR94] Smith KM, Abrahams PW, Dagleish MP, Steigmajer J (2009). The intake of lead and associated metals by sheep grazing mining-contaminated floodplain pastures in mid-Wales, UK: I. Soil ingestion, soil–metal partitioning and potential availability to pasture herbage and livestock. Science of The Total Environment.

[CR95] Sobańska MA (2005). Wild boar hair (*Sus scrofa*) as a non-invasive indicator of mercury pollution. Science of The Total Environment.

[CR96] Stamenkovic J, Gustin MS (2009). Nonstomatal versus Stomatal Uptake of Atmospheric Mercury. Environmental Science and Technology.

[CR97] Strom SM (2008). Total Mercury and Methylmercury Residues in River Otters (*Lutra canadensis*) from Wisconsin. Archives of Environmental Contamination and Toxicology.

[CR98] Szkoda J, Żmudzki J (2001). Toxic elements in tissues of game animals. Medycyna Weterynaryjna.

[CR99] Szkoda J, Durkalec M, Kolacz R, Opaliński S, Żmudzki J (2012). Content of cadmium, lead and mercury in the tissues of game animals. Medycyna Weterynaryjna.

[CR100] Szprengier T (1976). Mercury levels in the muscles and kidneys of horses, cows, and pigs. Bulletin of the Veterinary Institute in Pulawy.

[CR101] UNEP. 2013. Minamata Convention on mercury. http://www.mercuryconvention.org

[CR102] UNEP (2019). Global Mercury Assessment 2018.

[CR28] US EPA. 1992. Water Quality Standards, Establishment of Numeric Criteria for Priority Toxic Pollutants, States Compliance, Final Rule. Fed. Regist. 40 CFR Part 131, 57/246.

[CR103] Wang Y, Xie Q, Xu Q, Xue J, Zhang C, Wang D (2019). Mercury bioaccumulation in fish in an artificial lake used to carry out cage culture. Journal of Environmental Sciences.

[CR104] Wängberg I, Munthe J, Pirrone N, Iverfeldt A, Bahlman E, Costa P, Ebinghaus R, Feng X (2001). Atmospheric mercury distribution in northern Europe and in the Mediterranean region. Atmospheric Environment.

[CR105] Wyrzykowska B, Falandysz J, Jarzyńska G (2012). Metals in edible fish from Vistula River and Dead Vistula River channel, Baltic Sea. Journal of Environmental Science and Health, Part B.

[CR106] Yin RW, Zhang G, Sun Z, Feng JP, Hurley L, Yang L. Shang, Feng X (2017). Mercury risk in poultry in the Wanshan Mercury Mine, China. Environmental Pollution.

[CR107] Zhang Y, Jacob DJ, Horowitz HM, Chen L, Amos HM, Krabbenhoft DP, Slemr F, St VL, Louis, (2016). Observed decrease in atmospheric mercury explained by global decline in anthropogenic emissions. Proceedings of the National Academy of Sciences USA.

