# Supplementary material for: Status and trends of mercury pollution of the atmosphere and terrestrial ecosystems in Poland
Source: Ambio. 2021 Mar 23;50(9):1698–717. doi: 10.1007/s13280-021-01505-1 (PMC8285459; doi:10.1007/s13280-021-01505-1)
Supplement: Supplementary file 1 — Supplementary file1 (PDF 956 KB) [file 13280_2021_1505_MOESM1_ESM.pdf]

Ambio

ELECTRONIC SUPPLEMENTARY MATERIAL

This supplementary material has not been peer reviewed.

Title: **Status and trends of mercury pollution of the atmosphere and terrestrial ecosystems in Poland**

Authors: Agnieszka Jędruch, Lucyna Falkowska, Dominika Saniewska, Maciej Durkalec, Agnieszka Nawrocka, Elżbieta Kalisińska, Artur Kowalski, and Józef M. Pacyna (✉ lucyna.falkowska@ug.edu.pl, lucynafalkowska@gmail.com)

Extended Materials and Methods

Data collection

As already stated in the article, the first results on Hg level, both in the case of terrestrial and freshwater ecosystems, date back to the 1970s. However, most of the results from that period are very difficult to access as the journals and reports publishing them have not been digitized. The uncertainty of the archival data is also related to the capabilities of analytical methods used in that time. The methods commonly used in the 1970s and 1980s detected Hg at part per million (ppm) level and, as shown in Sect. Hg level in abiotic environmental compartments and Sect. Hg level in terrestrial plants and animals of the paper, the Hg concentrations in the investigated elements of Polish environment are generally much lower.

As the toxicity and bioavailability of Hg are dependent on its speciation, to fully assess the risk to wildlife and humans, it is important to consider the form of Hg in the environment. Although methylmercury (MeHg) is one of the most poisonous among Hg compounds, this study focuses only on THg. This It is because the knowledge of Hg speciation, both in abiotic and biotic environmental compartments in Poland, is mostly based on very highly limited data.

To determine the status and the temporal trends of THg pollution in Poland, a total of 85 data sources were used in this work, including 68 peer-reviewed journal articles, 3 books or book chapters, 6 reports, 6 legal acts, 1 thesis, and 1 open-access web material. These works were published between 1975 and 2020, with 84% of them after the year 2000 (**Fig. S1**). The discussion of data concerning Hg in the Polish ecosystem was based on additional 163 literature sources, including 151 peer-reviewed scientific papers, 5 books or book chapters, 3 conference proceeding articles, 3 reports, and 1 open-access web material published in years 1978-2020. As in the case of studies on Hg in Poland, the majority of works relating to Hg in other regions have been published in 2000 or later (**Fig. S1**). Due to the arrangement of the article, some of these references are provided here, in the Electronic Supplementary Material.

Data treatment

In this paper, the THg concentrations were presented in the following units: ng per m^3 for air, ng per L for water, ng per g of dry weight (dw) for soil, plants and mushrooms, and ng per g of wet weight (ww) for animal tissues and organs. In case the source data were in a different format, adequate conversion factors were applied. The converted values are marked with a symbol (*) and the calculation methods with references are given under each table.

Statistical analysis was carried out using the STATISTICA 12 (StatSoft). To model the relationship between variables analysed the linear regression was applied. This method was also used to determine the temporal changes of THg concentration, based on average values for each year. Considering the non-normal distribution of data and their limited number, to determine the significance of differences for data on Hg concentration obtained in the two periods, varying in Hg emission (before and after the year 2002 (**Fig. 1**, **Fig. S2**)), the nonparametric Mann-Whitney Test was used. The hypotheses were tested at a statistical significance level of $p=0.05$.

Figures presented in this study, including **Fig. 1**, **Fig. 3**, **Fig. 5**, **Fig. 6**, **Fig. 7**, **Fig. S1** and **Fig. S2**, were created using MS Excel and CorelDRAW X6. For the preparation of **Fig. 2** and **Fig. 4**, the ArcMap 10.4.1 (ESRI) was used, with the WGS 1984 coordination system and the UTM zone 34N for data projection. **Fig. 2** was prepared by the manual digitisation of the analogue map by Pasieczna (2012), with symbols as in the source. The **Fig. 4**, was created based on the spatial interpolation of point data using the inverse distance weighting (IDW) method (Bartier and Keller, 1996) and the geometrical intervals for symbol classification.

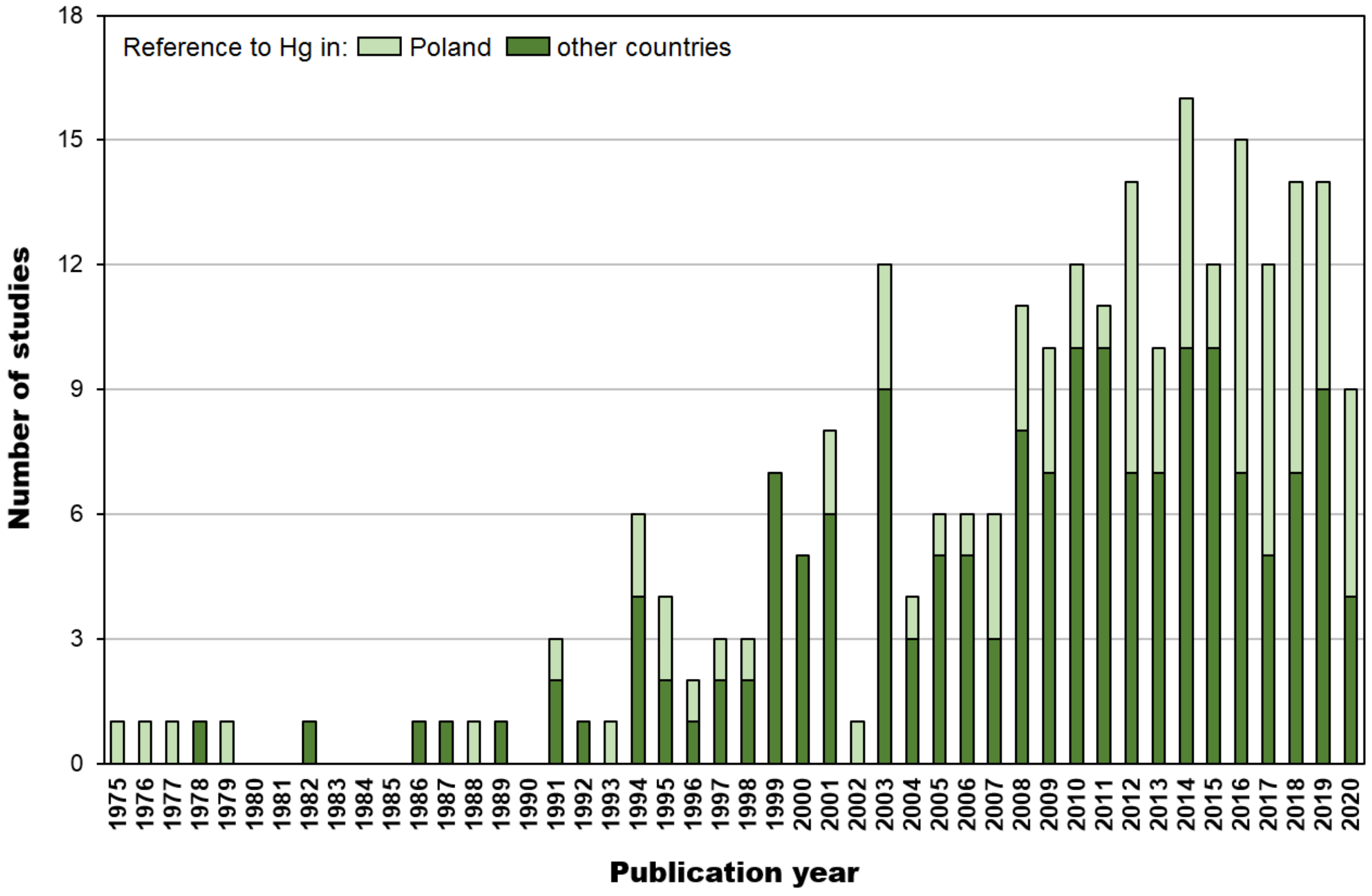

Figure S1 Number and publication year of studies cited in this work. The total number of references in this article, including the supplementary material, is 248

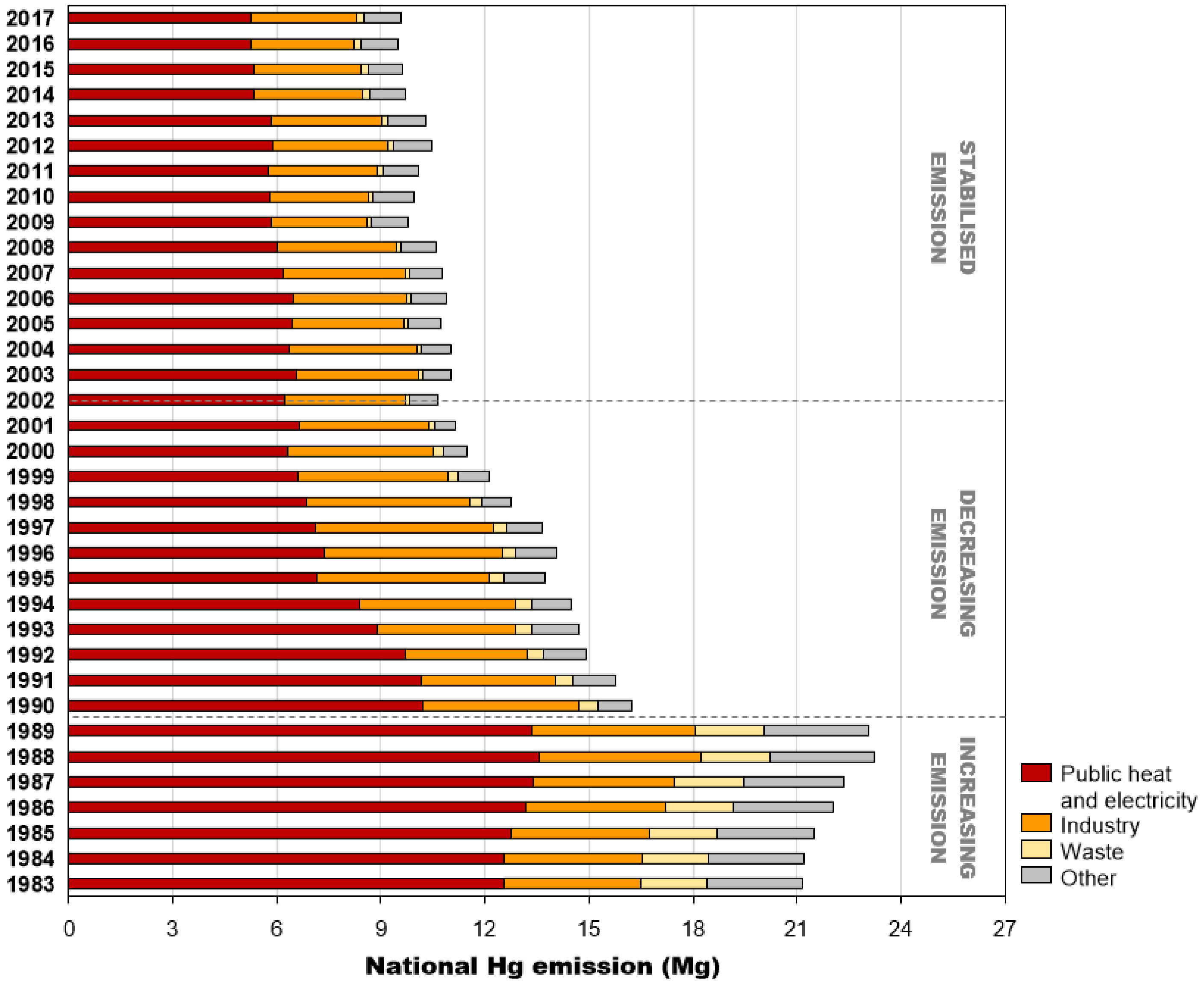

Figure S2 Annual total Hg emission (Mg) from the territory of Poland in the years 1983-2017. Data for 1983-1989 were estimated based on the Hg emission from Eastern Europe and the former USSR published by Pirrone et al. (1996) with the accuracy from 94 to 107%. Data for 1990-2017 were published by the KOBiZE (2019)

Table S1 Total Hg concentration (ng L^{-1}) in unfiltered water of rivers and streams in Poland and other regions of the world

	Region	Country	Study area	Period	Mean	Range	Reference
**A)BASE FLOW**							
	Europe	Spain	Valdeazogues (P)	1995-1997		LOD-23300	Berzas-Nevado et al., 2003
			Jarama (U)	2002-2004	10	8.1-15	Berzas-Nevado et al., 2008
			Tagus (U)	2002-2004	8.3	4.0-12.1	Berzas-Nevado et al., 2008
		Russia	Dvina	2011	48	2-95	Ovsepyan et al., 2016
		**Poland**	**Warta (P)**	**2003-2004**	**40**		Kowalski et al., 2007
			**Warta (U)**	**2003-2004**	**27**	**20-36**	Kowalski et al., 2007
			**Warta (U)**	**2003-2004**	**27**	**20-36**	Kowalski et al., 2007
			**Cybina (U)**	**2003-2004**	**12**		Kowalski et al., 2007
			**Odra**	**2014**	**12**		HELCOM, 2018 *
			**Kacza**	**2012-2013**	**6.7**	**0.7-10.6**	Gębka et al., 2018
				**2008-2009**	**2.9**	**0.2-14.7**	Saniewska et al., 2014b
			**Vistula**	**2012**	**7.4**		Bełdowska et al., 2016
				**2010**	**7.3**	**4.7-11.7**	Saniewska et al., 2014a
				**2014**	**6.7**		HELCOM, 2018 *
			**Gizdepka**	**2012-2013**	**5.2**	**0.1-14.4**	Gębka et al., 2018
			**Zagórska Struga**	**2012-2013**	**4.9**	**1.2-19.8**	Gębka et al., 2018
			**Reda**	**2012-2013**	**4.1**	**1.4-17.3**	Gębka et al., 2018
			**Płutnica**	**2010**	**4.1**	**1.0-16.0**	Saniewska, 2013
			**Oliwski Stream**	**2008-2009**	**2.8**	**0.5-9.3**	Saniewska et al., 2014b
		Estonia	Pärnu	2014	11.1		HELCOM, 2018 *
		Finland	Tornionjoki	2014	4.1		HELCOM, 2018 *
			Kemijoki	2014	2.7		HELCOM, 2018 *
			Kokemäenjoki	2014	2.6		HELCOM, 2018 *
			Oulujoki	2014	1.7		HELCOM, 2018 *
			Kymijoki	2014	1.7		HELCOM, 2018 *
		Lithuania	Nemunas	2014	2.8		HELCOM, 2018 *
		Sweden	various watercourses	2000-2010		0.5-4.5	Eklöf et al., 2012
				2016	0.5	0.2-0.9	Bravo et al., 2018
			Dalälven	2014	2.2		HELCOM, 2018 *
			Ljusnan	2014	2.1		HELCOM, 2018 *
			Ångermanälven	2014	1.6		HELCOM, 2018 *
			Lule älv	2014	1.5		HELCOM, 2018 *
			Göta älv	2014	1.4		HELCOM, 2018 *
			Kalix älv	2014	1.3		HELCOM, 2018 *
			Ume älv	2014	1.0		HELCOM, 2018 *
			Indalsälven	2014	0.8		HELCOM, 2018 *
		Latvia	Slocene	2005-2011	1.4		Bogans et al., 2011
			Lielupe	2005-2011	0.4		Bogans et al., 2011
		UK	various watercourses	2016	1.5	0.3-2.8	Bravo et al., 2018
		Czech Republic	various watercourses	2016	0.8		Bravo et al., 2018
		France	various watercourses	2016	0.7	0.5-0.9	Bravo et al., 2018
		Austria	various watercourses	2016	0.6	0.1-1.0	Bravo et al., 2018
		Germany	various watercourses	2016	0.4	0.3-0.6	Bravo et al., 2018
		Spain	various watercourses	2016	0.4	0.1-0.9	Bravo et al., 2018
		Bulgaria	various watercourses	2016	0.3		Bravo et al., 2018
	North America	USA	San Carlos Creek (P)	1997-1999		640-12400	Ganguli et al., 2000
			Kuskokwim (P)			1 800-2500	Gray et al., 2000
			Hudson River (U)	2001	62	28-116	Heyes et al., 2004
			Ohio	2009		0.6-37	Naik and Hammerschmidt, 2011
			San Carlos Creek	1997-1999		4.2-12	Ganguli et al., 2000
			Provo	2015-2017	3.1	0.3-8.2	Packer et al., 2020
			Herring Run (U)	1997	2.4		Lawson et al., 2001
			Choptank	1997	2.4		Lawson et al., 2001
			Potomac	1997	2.4		Lawson et al., 2001
			Rappahannock	1997	2.1		Lawson et al., 2001
			Susquehanna	1997	1.4		Lawson et al., 2001
			Patapsco	1997	1.4		Lawson et al., 2001
		Canada	Yukon	2001-2005	15		Schuster et al., 2011
	South America	Brasil	Xingu (P)	2014		LOD-220	Gomes Ribeiro et al., 2017
			Tapajós	2013	4.6	0.7-23.8	Lino et al., 2019
		Bolivia	Coroico	1995-1996		2.9-9.6	Maurice-Bourgoin et al., 1999
			Zongo	1995-1996		7.2-8.2	Maurice-Bourgoin et al., 1999
			Beni	1995-1996		2.2-2.6	Maurice-Bourgoin et al., 1999

Table S1 Continued

	Region	Country	Study area	Period	Mean	Range	Reference
	Asia	China	Xiaxihe (P)	2000		550-10580	Horvat et al., 2003
			Wuli (U)	2007	920	210-2700	Wang et al., 2009
			Meizixi (P)	2002	586		Feng et al., 2003
			Lianshan (U)	2007	260	230-290	Wang et al., 2009
			Xiaxihe	2000	130	20-310	Horvat et al., 2003
			Yantgze	2013	90	50-160	Wu, 2014
			Cishan (U)	2007	84		Wang et al., 2009
			Dongjiang	2009	19	11-49	Liu et al., 2012
		China, Nepal, India	Koshi	2017-2019	5.8	0.6-33	Sun et al., 2020
		Indonesia	Kahayan (P)	2004-2007		20-2260	Elvince et al., 2008
			Rungan	2004-2007		16-117	Elvince et al., 2008
**B)HIGH FLOW**							
	Europe	**Poland**	**Poland, Vistula (flood)**	**2010**	**59**	**21-299**	Saniewska et al., 2014a
	North America	USA	Potomac	1997	18.6		Lawson et al., 2001
			Herring Run (U)	1997	12.6		Lawson et al., 2001
			Provo (snowmelt)	2015-2017	8.9	3.7-19.4	Packer et al., 2020
			Susquehanna	1997	6.6		Lawson et al., 2001
			Patapsco	1997	5.2		Lawson et al., 2001
			Rappahannock	1997	5.0		Lawson et al., 2001
			Choptank	1997	3.1		Lawson et al., 2001
	Asia	China, Nepal, India	Koshi River (monsoon)	2017-2019	6.7	0.7-27	Sun et al., 2020

P – polluted area, U – urban area

* Values calculated on the basis of the annual Hg load and average river flow.
Data were obtained through national monitoring programmers and reported by Contracting Parties to HELCOM in the frame to the Sixth Baltic Sea Pollution Load Compilation project (PLC-6). In accordance to EU Water Frame Directive (EC, 2013), heavy metals can be reported as dissolved concentrations, i.e. the dissolved phase of a water sample obtained by filtration through a 0.45 μm filter or any equivalent pre-treatment (HELCOM, 2015).

Table S2 Total Hg concentration (ng g^{-1} dw) in terrestrial vegetation: **a)** moss, **b)** grass, and **c)** caps of edible mushroom from Poland and other regions of the world

	Region	Study area	Period	Mean	Range	Reference
**A) MOSS**						
Mixed species	Europe	Slovakia	2000	180	<840	Harmens et al., 2008
		Lithuania	2000	88	62-114	Harmens et al., 2008
		France	2000	70	42-98	Harmens et al., 2008
		Italy	2000	70	<492	Harmens et al., 2008
		**Poland, Holy Cross Mountains**	**2008**	**55**	**28-97**	Migaszewski et al., 2010
		Norway	2000	52	30-74	Harmens et al., 2008
		Russia	1995	50	<132	Harmens et al., 2008
		Latvia	2000	50	29-71	Harmens et al., 2008
		Austria	2000	50	20-80	Harmens et al., 2008
		Czech Republic	2000	48	33-63	Harmens et al., 2008
				44	22-150	Suchara et al., 2011
		Finland	2000	42	19-61	Harmens et al., 2008
		Germany	2000	41	19-63	Harmens et al., 2008
		Iceland	2000	39	18-60	Harmens et al., 2008
		Ukraine	2000	39	17-61	Harmens et al., 2008
		Switzerland	2000	32	21-43	Harmens et al., 2008
		**Poland, Sudety Mountains**	**2010**	**18**	**12-35**	Kłos et al., 2012
		**Poland, Baltic coast**	**2012**	**17**	**11-23**	Bełdowska et al., 2016
		Sweden	2000	17	1-36	Harmens et al., 2008
	North America	USA, Pennsylvania	2000-2005	424	300-580	Davis et al., 2007
		Canada, Kejimkujik Park	1997		45-395	Rencz et al., 2003
		Greenland		108	59-196	Riget et al., 2000
		USA, Alaska	2014-2016	58	52-64	Olson et al., 2019
			2007	52	20-101	Migaszewski et al., 2010
		Canada, Yukon	2010	41	24-97	Rempel, 2010
	Asia	China, Guizhou (P)	2002	16212	980-95000	Qiu et al., 2005
		China, Mt. Gongga	1990	90	85-95	Wang et al., 2019
		China, Tibetan Plateau	2010-2012	54	13-273	Shao et al., 2017
	Antarctica	Victoria Land	2002	180	27-570	Bargagli et al., 2005
		Deception Island	2011	43	27-56	Mão de Ferro, et al., 2014
**B) GRASS**						
Mixed species	Europe	Spain, Almaden (P)			740-28100	Molina et al. 2006
		Sweden	1990-1994	66	36-98	Xiao et al., 1998
		**Poland, LGCD (P)**		**39**	**4-69**	Barej, et al., 2009
		**Poland, Karkonosze Mountains**		**30**	**19-53**	Barej, et al., 2009
		Czech Republic		13	5-29	Suchara et al., 2011
		**Poland, Baltic coast**	**2012**	**12**	**10-16**	Bełdowska et al., 2016
	North America	USA, Alaska	2014-2016	9	8-10	Olson et al., 2019
	South America	Bolivia, ASGM (P)	2009	6300	3000-10400	Terán-Mita et al., 2013
		Brasil, Tapajós (P)		242	20-480	Egler et al., 2006
	Africa	Ghana, Obuasi (P)	1992-1993	2580	200-6200	Amonoo-Neizer et al., 1996
**C) MUSHROOMS**						
King bolete						
(*Boletus edulis*)	Europe	Slovenia, Idrja (P)	2014-2016	69000	54200-83800	Kavčič et al., 2019
		Slovakia (P)		16260	4940-35870	Musilová et al., 2019
		**Poland, Holy Cross Mountains**	**2000**	**7600**	**4000-14000**	Falandysz et al., 2007
		Slovenia		5790	4430-7150	Kavčič et al., 2019
		Slovakia		4087	624-23390	Musilová et al., 2019
		Czech Republic	2001-2003	3295	2000-6100	Nováčkova et al., 2007
		Italy, Tuscany	2008-2009	2800	1000-6100	Giannaccini et al., 2012
		Italy, Emilia		2670	1020-4320	Cocchi et al., 2006
		**Poland, NE**	**1996-2000**	**2530**	**200-8600**	Falandysz et al., 2007
		**Poland, NW**	**2009**	**2340**	**2120-2500**	Mazurkiewicz and Podlasińska, 2014
		**Poland, Tatry Mountains**	**2000**	**2300**	**670-4300**	Falandysz et al., 2007
		Croatia	2013	2180	730-5710	Širić et al., 2015
		**Poland, Sudety Mountains**	**2000**	**2100**	**1300-3100**	Falandysz et al., 2007
		Spain	2005-2006	2000	800-3200	Melgar et al., 2009
		Sweden	1995	1200	58-5400	Falandysz et al., 2001
	Asia	China, Yunnan	2011-2014	4500	1600-22000	Falandysz et al., 2015
		China, Sichuan	2000-2011	4000	1600-7500	Zhang et al., 2010

P – polluted area

Table S3 Total Hg concentration (ng g^{-1} ww) in terrestrial animals: **a)** freshwater fish, **b)** wild animals, and **c)** livestock from Poland and other regions of the world

	Region	Study area	Period	Muscle Mean	Muscle Range	Liver Mean	Liver Range	Reference
**A) FRESHWATER FISH**								
Common bream								
(*Abramis brama*)	Europe	Czech Republic	2003	960	550-1230	1500	630-2980	Marsálek et al., 2005
			1991-1996	298	108-597			Dušek et al., 2005
		Finland	1972-1975	340	50-810			Hattula et al., 1978
		France	2007-2013	194	171-246			Nguetseng et al., 2015
			2000-2008	128	65-241			Noël et al., 2013
			2009-2010	36	19-46	35	7-63	Gentès et al., 2013 *
		Slovenia	2006	160	50-480			Mazej et al., 2010
		Netherlands	2007-2013	158	103-236			Nguetseng et al., 2015
		Sweden	2007-2013	150	123-180			Nguetseng et al., 2015
		**Poland**	**2011-2013**	**87**	**1-391**			Szkoda et al., 2014
		UK	2007-2013	51	43-60			Nguetseng et al., 2015
		Germany	2007-2013	26	18-43			Nguetseng et al., 2015
			1989-1991	19	3-47			Scharenberg et al., 1994
		Hungary	1999-2000	25	15-37	23	5-62	Farkas et al., 2003 *
	Asia	Russia		400	50-2600			Koval et al., 1999
Pike								
(*Esox lucius*)	Europe	Finland	1972-1975	1070	230-3690			Hattula et al., 1978
			2009-2013	290	118-590			Ahonen et al., 2018 *
		Sweden	1994-2006	560	160-2400			Åkerblom et al., 2012
			1974-1999		350-1390			Lindeström, 2001
		France	2009-2010	544	353-736			Gentès et al., 2013 *
			2000-2008	162	92-232			Noël et al., 2013
		UK (P)	1995-1996	342	252-432			Yamaguchi et al., 2003
		Germany	1993-1994		220-850		<450	Meinelt et al., 1997
		**Poland**	**2011-2013**	**229**	**1-400**			Szkoda et al., 2014
		Hungary	2000		150-303			Fleit and Lakatos, 2003
		Czech Republic	2005	180				Kensova et al., 2010
	North America	Canada	1978-2004	666	70-4110			Lavigne te al., 2010
		USA, Alaska	2000		628-1506			Jewett et al., 2003
		USA, Royale Is.	2004-2006	122-299	69-622	87-392	48-3074	Drevnick et al., 2008
	Asia	Iran	2011	24-48	21-84			Zamani-Ahmadmahmoodi et al., 2014
Common roach								
(*Rutilus rutilus*)	Europe	Czech Republic	2003	810	580-1270	880	390-1690	Marsálek et al., 2005
			1991-1996	278	89-445			Dušek et al., 2005
		Finland	1972-1975	500	110-1130			Hattula et al., 1978
			2009-2013	100	53-150			Ahonen et al., 2018 *
		Slovakia	2004	310	200-380			Andreji et al., 2005
		Germany	2008-2015	165	39-332			Lepom and Wellmitz, 2017
			1993	59	16-169			Falter and Schöler, 1994
		Sweden	1991-1993	130	20-540			Sonesten, 2001
		France	2000-2008	94	36-299			Noël et al., 2013
			2009-2010	81	35-123	87	10-597	Gentès et al., 2013 *
		Slovenia	2006	80	30-160			Mazej et al., 2010
		UK (P)	1995-1996	76	42-101			Yamaguchi et al., 2003
		UK	1991-1994	54	19-99			Edwards et al., 1999
		**Poland**	**2014**	**72**	**58-102**	**38**	**20-52**	Łuczyńska et al., 2018
	Asia	Russia		220	50-700			Koval et al., 1999
		Iran		45	22-65			Zolfaghari, 2018 *
**B) WILD ANIMALS**								
Red deer								
(*Cervus elaphus*)	Europe	Croatia	2002-2005	6		9		Lazarus et al., 2008
			1990-2012	3.7		7.4		Lazarus et al., 2014
			2007-2008		0.6-1.1		2.2-5.1	Srebočan et al., 2012
		**Poland (P)**	**2009-2010**	**1.8-5.4**	**0.1-8.6**	**6.7-17.2**	**2.6-10.6**	**Albińska et al., 2011**
		**Poland**	**2013-2014**	**1.0**	**0.1-2.0**	**7.0**	**0.1-48.0**	**Giżejewska et al., 2014**
		Spain	2004-2006	0.3	0.1-2.8	3.6	0.1-16.9	Berzas Nevado et al., 2012
Roe deer								
(*Capreolus capreolus*)	Europe	Hungary		870	240-1460			Lehel et al., 2015
		Slovenia (P)	1991-1997	79	28-143	849	295-2270	Gnamuš et al., 2000

Table S3 Continued

	Region	Study area	Period	Muscle<br>Mean	Range	Liver<br>Mean	Range	Reference
		Slovakia	1991			27	2-132	Findo et al., 1993
		Austria	1995			12	0-36	Gufler et al., 1997
			2012-2013	<4				Ertl et al., 2016
		**Poland (P)**	**2011-2013**	**3**	**0.1-12**	**9**	**0.1-61**	Durkalec et al., 2015
		Slovenia	1991-1997	2.8	2-5	14	10-17	Gnamuš et al., 2000
		Croatia	1990-2012	1.5		9		Lazarus et al., 2014
		**Poland**	**2011-2013**	**1**	**0.1-9**	**5.0**	**0.1-25.0**	Durkalec et al., 2015
		Serbia	2013-2014	<1				Baloš et al., 2015
		Czech Republic	2005-2007	0.6	0.5-3.5	11	3-41	Čelechovská et al., 2008
Wild boar								
(*Sus scrofa*)	Europe	Serbia	2004-2005				6-388	Petrović, 2007
		Slovakia	2009-2010	30		40		Gasparik et al., 2012
		Italy	1992	10	9-12			Barghigiani and Ristori, 1994
		Croatia	1990-2012	9		53		Lazarus et al., 2014
			2008-2009	4-12	1-61			Bilandžić et al., 2010b
			2008-2009	4-10	1-125	12-27	1-146	Florijančić et al., 2015
		Austria	2012-2013	8				Ertl et al., 2016
		**Poland (P)**	**2011-2013**	**7**	**0.1-23**	**26**	**6-84**	Durkalec et al., 2015
		**Poland**	**2011-2019**	**6**	**0.1-20**	**19**	**3-28**	Durkalec et al., 2015
		Spain (P)	2005			18	<96	Berzas Nevado et al., 2012
		Spain	2005	6	8-103	8	0.2-41	Berzas Nevado et al., 2012
Red fox								
(*Vulpes vulpes*)	Europe	Spain	2004-2006	120		283		Millán et al., 2008
		Russia (P)	2007-2011	90	20-160	310	150-470	Komov et al., 2016
		**Poland**	**2004-2006**	**70**	**20-240**	**140**	**40-520**	Kalisińska et al., 2012
		Russia	2015-2018	44	12-83	107	22-204	Khabarova et al., 2018
		Croatia	2008-2009	29	4-82	89	11-288	Bilandžić et al., 2010a
		Slovakia	1998-1999	13	1-32	220	1-450	Piskorová et al., 2003
		Italy (P)	1992			160	20-300	Corsolini et al., 1999
		Italy	1994-1995			30	0-620	Alleva et al., 2006
	North America	Canada	1997-1998	220		410		Champoux et al., 1999
		USA, Alaska	2010-2011	157	27-556	341	32-1708	Dainowski et al., 2015
		USA, Wisconsin	1972-1975	20		40		Sheffy and Amant, 1982
**C) LIVESTOCK**								
Cattle	Europe	Czech Republic	2005-2007	21		24		Čelechovská et al., 2008
		Finland	1987-1988	12		11		Niemi et al., 1991
		Sweden	1984-1988	5	3-17	6	3-26	Jorhem et al., 1991
		Slovakia	2011-2012	2.7	1.6-3.8			Lukáčová et al., 2014
		Netherlands	1980-1985	1	0-16	3	7-14	Vos et al., 1987
		**Poland**	**2009-2018**	**0.8**	**0.5-53**	**1.8**	**0.5-51**	Nawrocka et al., 2020
		Spain	1996-1997	0.5	0-19	1	0-94	Alonso et al., 2003
	South America	Brazil	2011	12	0.3-40			Batista et al., 2012
	Asia	Saudi Arabia	2011	16	9-24			Alturiqi and Albedair, 2012 *
		Iran		3	0-170	2	0-11	Hashemi, 2018
		Pakistan		0.6	0.4-0.9	0.3	0.2-0.4	Mariam et al., 2004
	Africa	Ghana	2012	14	12-17			Nkansah and Ansah, 2014 *
		Tanzania	2005	11-16	10-81	49-112	10-436	Chibunda and Janssen, 2009
		Algeria	2012	11	9-14			Badis et al., 2014 *
		Egypt		4	3-5	6	5-7	Khallafalla et al., 2011
Pigs	Europe	Finland	1987-1988	11		12		Niemi et al., 1991
		Sweden	1984-1988	9	3-30	15	6-54	Jorhem et al., 1991
		Netherlands	1980-1985	2	1-20	2	1-29	Vos et al., 1986
		Slovakia	2011-2012	1.3	0.8-1.8			Lukáčová et al., 2014
		Spain	2004	1	0.15-5	1	0.15-4	López-Alonso et al., 2007
		**Poland**	**2009-2018**	**0.7**	**0.5-36**	**1.2**	**0.5-108**	Nawrocka et al., 2020
		France	2014		<LOD		<LOD	Parinet et al., 2018
	South America	Brazil	2011	23	0.5-130			Batista et al., 2012
Chicken	Europe	Turkey					39-84	Demibraş, 1999
		**Poland**	**2003**	**3**	**1-11**	**4**	**1-12**	Opaliński et al., 2004
			**2014-2015**	**0.7**	**0.1-1.8**	**2.5**	**0.4-16**	Dobrzański et al., 2017
		Greece			0.1-3.1		0.5-16.5	Kambamanoli-Dimou et al., 1989
		Romania					<2.8	Ghimpeteanu et al., 2012

Table S3 Continued

Region	Study area	Period	Muscle Mean	Muscle Range	Liver Mean	Liver Range	Reference
South America	Brazil	2011	8.4	0.5-30			Batista et al., 2012
Asia	Saudi Arabia	2011	3.2	2.3-3.8			Alturiqi and Albedair, 2012 *
	Pakistan	2008-2009	0.5	0.4-0.7	0.8	0.4-1	Shah et al., 2010 *
	Indonesia		0.6				Surtipanti et al., 1995
Africa	Algeria	2012	2.3-3.8				Badis et al., 2014 *

P – polluted area

* Values converted from dry weight
To convert the THg concentration in dry weight (dw) to wet weight (ww) the following conversion factors were applied: fish: 4.0 (Nogueira et al., 2013); cattle: 3.6 (Olsson et al., 2001); chicken: 3.9 (Mazzoni et al., 2015)

Table S4 Total Hg concentration in muscles and livers of terrestrial animals with different feeding habits: **a)** freshwater fish, **b)** wild animals, and **c)** livestock (ng g^{-1} ww) from Poland (data are also presented in the **Fig. 5**)

	Feeding habit	Period	Muscle<br>Mean	 Range	Liver<br>Mean	 Range	Reference
**A) FRESHWATER FISH**							
Common rudd (*S. erythrophthalmus*)	planktivorous	2009-2014	92	66-109			Kalisińska et al. 2017
Vendace (*C. albula*)	planktivorous	2010	114	50-139			Łuczyńska et al., 2016
European whitefish (*C. lavaretus*)	benthivorous	2010	65	54-81			Łuczyńska et al., 2016
Common roach (*R. rutilus*)	benthivorous	2014	72	58-102	38	20-52	Łuczyńska et al., 2018
Common bream (*A. brama*)	benthivorous	2011-2013	87	<391			Szkoda et al., 2014
White bream (*B. bjoerca*)	benthivorous	2009-2014	132	96-148			Kalisińska et al. 2017
Crucian carp (*C. carassius*)	benthivorous	2000-2014	156	100-270			Wyrzykowska et al., 2012; Kalisińska et al. 2017
European eel	benthivorous		179	28-487			Polak-Juszczak and Nermer, 2016
Tench (*T. tinca*)	benthivorous	2000	340	220-520			Wyrzkowska et al., 2012
Burbot (*L. lota*)	piscivorous	2000	94	72-120			Wyrzkowska et al., 2012
Pike-perch (*S. lucioperca*)	piscivorous	2011-2013	153	<456			Szkoda et al., 2014
Perch (*P. fluviatilis*)	piscivorous	2014	162	98-259	58	43-94	Łuczyńska et al., 2018
Ide (*L. idus*)	piscivorous	2009-2014	189	185-228			Kalisińska et al. 2017
Pike (*E. lucius*)	piscivorous	2011-2013	229	<400			Szkoda et al., 2014
**B) WILD ANIMALS**							
European rabbit (*O. cuniculus*)	herbivorous	1988-1989	1	<5	2	1-3	Żarski et al., 1995a
European bison (*B. bonasus*)	herbivorous	2012-2016			3	1-6	Durkalec et al., 2018
Red deer (*C. elaphus*)	herbivorous	2013-2014	1	<2	7	<48	Giżejewska et al. 2017
Roe deer (*C. capreolus*)	herbivorous	2011-2013	1	<9	5	<25	Durkalec et al. 2015
European hare (*L. europeaus*)	herbivorous	1988-1989	8	3-13	22	1-43	Żarski et al., 1995b
Eurasian beaver (*C. fiber*)	herbivorous	2011	11	10-11	24	18-42	Gizejewska et al., 2014
Wild boar (*S. scrofa*)	omnivorous	2011-2013	6	<20	19	3-28	Durkalec et al. 2015
Red fox (*V. vulpes*)	omnivorous	2004-2006	70	20-240	140	40-520	Kalisińska et al. 2012b
Racoon (*P. lotor*)	omnivorous	2009-2014	152	3-730			Kalisińska et al. 2017
Eurasian otter (*L. lutra*)	piscivorous	2009-2014	972	508-1601			Kalisińska et al. 2017
American mink (*N. vison*)	piscivorous	2009-2011	2801	1781-4178	3650	1020-6470	Kalisińska et al., 2012a
**C) LIVESTOCK**							
Rabbit		1987	1.0	<2.0	3.0	1.0-4	Falandysz, 1991
Cattle		2009-2018	1.0	0.5-7	1.8	0.5-14	Nawrocka et al., 2020
Sheep		2002-2006	1.9	1.0-14.5	3.1	1.0-15.8	Zięba, 2003; Rudy, 2009
Pig		2009-2018	0.7	0.5-16.4	1.2	0.5-24.1	Nawrocka et al., 2020
Chicken		2014-2015	0.7	<1.8	2.5	<16	Dobrzański et al., 2017
Geese		1987	1.0	<4.0	4.0	1.0-7.0	Falandysz, 1991
Duck		1987	3.1	1.0-7.0	10.0	5.0-17	Falandysz, 1991
Turkey		1987	6.0	3.0-9.0	8.0	5.0-10	Falandysz, 1991

Table S5 Temporal changes of total Hg concentration in muscles and livers of: **a)** wild animals, and **b)** livestock (ng g^{-1} ww) from Poland (data are also presented in the **Fig. 6** and the **Fig. 7**)

	Year	Muscle Mean	Range	N	Liver Mean	Range	N	Reference
**A)WILD ANIMALS**								
Wild boar								
(*Sus scrofa*)	1988	5	1-23	118	18	5-42	57	Falandysz, 1994
	1989	3	1-9	116	13	7-23	10	Falandysz, 1994
	1990	2	1-5	119	8	5-12	12	Falandysz, 1994
	1991	2	1-6	69				Falandysz, 1994
	1998	7	<32	186				Szkoda and Żmudzki, 2001
	1999	6	<30	150	20		460	Szkoda and Żmudzki, 2001
	2000	7	<39	124	17	4-25	45	Szkoda and Żmudzki, 2001; Sobańska, 2005
	2001				36	15-61	14	Dobrowolska and Melosik, 2002
	2009	5	1-30	59	12	1-26	40	Nawrocka et al., 2020
	2010	8	3-15	13	20	14-32	12	Nawrocka et al., 2020
	2011	8	1-215	63	15	1-40	47	Nawrocka et al., 2020
	2012	4	1-9	27	17	1-37	26	Nawrocka et al., 2020
	2013	5	1-15	51	19	1-142	48	Nawrocka et al., 2020
	2014	7	1-26	59	14	1-42	53	Nawrocka et al., 2020
	2015	4	1-21	49	12	1-73	40	Nawrocka et al., 2020
	2016	4	1-23	70	18	3-76	50	Nawrocka et al., 2020
	2017	7	1-48	111	20	1-93	95	Nawrocka et al., 2020
	2018	5	1-29	98	16	1-83	65	Nawrocka et al., 2020
Cervids	1988	2	<18	171	10	2-65	77	Falandysz, 1994
	1989	1	<5	177	5	2-11	20	Falandysz, 1994
	1990	1	<3	165	7	2-23	20	Falandysz, 1994
	1991	1	<4	119				Falandysz, 1994
	1998	3	<28	205				Szkoda and Żmudzki, 2001
	1999	3	<32	222	7		616	Szkoda and Żmudzki, 2001
	2000	2	<19	189				Szkoda and Żmudzki, 2001
	2001				6		6	Dobrowolska and Melosik, 2002
	2009	1	<5	56	8	<32	37	Nawrocka et al., 2020
	2010	1	1-11	16	2	1-14	16	Nawrocka et al., 2020
	2011	1	1-3	20	3	1-16	20	Nawrocka et al., 2020
	2012	1	1-3	24	4	1-12	24	Nawrocka et al., 2020
	2013	1	1-11	31	2	1-10	26	Nawrocka et al., 2020
	2014	1	1-4	52	2	1-9	35	Nawrocka et al., 2020
	2015	1	1-28	55	4	1-41	41	Nawrocka et al., 2020
	2016	1	1-3	51	2	1-13	29	Nawrocka et al., 2020
	2017	1	1-3	60	2	1-20	65	Nawrocka et al., 2020
	2018	1	1-8	86	3	1-21	79	Nawrocka et al., 2020
**B)LIVESTOCK**								
Cattle	1988	2	1-5	58	5	2-15	58	Falandysz, 1993
	1989	1	<2	53	4	1-10	53	Falandysz, 1993
	1990	1	<3	44	5	1-66	44	Falandysz, 1993
	1991	1	<5	44	4	<21	44	Falandysz, 1993
	2009	2	1-7	4	1		1	Nawrocka et al., 2020
	2010	2	1-53	33	3	1-14	25	Nawrocka et al., 2020
	2011	1	1-9	49	3	1-23	29	Nawrocka et al., 2020
	2012	1	1-7	131	2	1-11	51	Nawrocka et al., 2020
	2013	1	1-7	83	2	1-12	37	Nawrocka et al., 2020
	2014	1	1-9	91	2	1-8	22	Nawrocka et al., 2020
	2015	1	1-4	59	1	1-5	50	Nawrocka et al., 2020
	2016	1	1-3	41	1	1-7	34	Nawrocka et al., 2020
	2017	1	1-10	155	1	1-11	215	Nawrocka et al., 2020
	2018	1	<1	170	2	1-51	175	Nawrocka et al., 2020
Pigs	1988	3	<18	324	5	1-29	324	Falandysz, 1993
	1989	1	<6	330	4	1-15	330	Falandysz, 1993
	1990	1	<5	280	3	<17	280	Falandysz, 1993
	1991	1	<3	247	3	1-11	246	Falandysz, 1993
	2009	1	1-4	17	2	1-10	12	Nawrocka et al., 2020
	2010	1	1-15	35	2	1-8	27	Nawrocka et al., 2020
	2011	1	1-4	96	1	1-4	58	Nawrocka et al., 2020
	2012	1	1-7	242	1	1-24	78	Nawrocka et al., 2020

Table S5 Continued

		Muscle			Liver			
	Year	Mean	Range	N	Mean	Range	N	Reference
	2013	1	1-36	183	1	1-10	46	Nawrocka et al., 2020
	2014	**1**	1-8	170	**1**	1-4	47	Nawrocka et al., 2020
	2015	**1**	1-3	123	**1**	1-6	87	Nawrocka et al., 2020
	2016	**1**	<1	101	**1**	1-15	105	Nawrocka et al., 2020
	2017	**1**	1-4	240	**1**	1-108	341	Nawrocka et al., 2020
	2018	**1**	1-16	234	**1**	1-24	273	Nawrocka et al., 2020

REFERENCES

Åkerblom, S., M. Nilsson, J. Yu, B. Ranneby, and K. Johansson. 2012. Temporal change estimation of mercury concentrations in northern pike (*Esox lucius* L.) in Swedish lakes. *Chemosphere* 86: 439-445. https://doi.org/10.1016/j.chemosphere.2011.09.037

Ahonen, S.A., B. Hayden, J.J. Leppänen, and K.K. Kahilainen. 2018. Climate and productivity affect total mercury concentration and bioaccumulation rate of fish along a spatial gradient of subarctic lakes. *Science of The Total Environment* 637-638: 1586-1596. https://doi.org/10.1016/j.scitotenv.2018.04.436

Albińska, J., J. Góralski, M.I. Szynkowska, E. Leśniewska, and T. Paryjczak. 2011. Mercury in carcasses of wild animals hunted in the province of Lodz. *Rocznik Ochrona Środowiska* 13: 525-538.

Alleva, E., N. Francia, M. Pandolfi, A.M. De Marinis, F. Chiarotti, and D. Santucci. 2006. Organochlorine and Heavy-Metal Contaminants in Wild Mammals and Birds of Urbino-Pesaro Province, Italy: An Analytic Overview for Potential Bioindicators. *Archives of Environmental Contamination and Toxicology* 51: 123-134. https://doi.org/10.1007/s00244-005-0218-1

Alturiqi, A.S., and L.A. Albedair. 2012. Evaluation of some heavy metals in certain fish, meat and meat products in Saudi Arabian markets. *Egyptian Journal of Aquatic Research* 38: 45-49. https://doi.org/10.1016/j.ejar.2012.08.003

Amonoo-Neizer, E.H., D. Nyamah, and S.B. Bakiamoh. 1996. Mercury and arsenic pollution in soil and biological samples around the mining town of Obuasi, Ghana. *Water, Air, and Soil Pollution* 91: 363. https://doi.org/10.1007/BF00666270

Andreji, J., I. Stránai, P. Massányi, and M. Valent. 2006. Accumulation of Some Metals in Muscles of Five Fish Species from Lower Nitra River. *Journal of Environmental Science and Health, Part A* 41: 2607-2622. https://doi.org/10.1080/10934520600928003

Badis, B., Z. Rachid, and B. Esma. 2014. Levels of Selected Heavy Metals in Fresh Meat from Cattle, Sheep, Chicken and Camel Produced in Algeria. *Annual Research and Review in Biology* 4: 1260-1267.

Baloš, M.Ž., Ž. Mihaljev, S. Jakšić, N. Prica, G. Lazić, M. Kapetanov, and J.P. Radulović. 2015. The Incidence Of Heavy Metals And Other Toxic Elements In Roe Deer (*Capreolus capreolus*) Tissues. *Archivos de Medicina Veterinaria* 8: 3-10.

Barej, R., R. Kwaśnicki, K. Chojnacka, J. Bolanowski, Z. Dobrzański, and P. Pokorny. 2009. Mercury Content in Rural and Industrial Regions in Lower Silesia. *Polish Journal of Environmental Studies* 18: 547-552.

Bargagli, R., C. Agnorelli, F. Borghini, and F., Monaci. 2005. Enhanced Deposition and Bioaccumulation of Mercury in Antarctic Terrestrial Ecosystems Facing a Coastal Polynya. *Environmental Science and Technology* 39: 8150-8155. https://doi.org/10.1021/es0507315

Barghigiani, C., and T. Ristori. 1994. Mercury Levels in Agricultural Products of Mt. Amiata (Tuscany, Italy). *Archives of Environmental Contamination and Toxicology* 26: 329-334. https://doi.org/10.1007/BF00203559

Bartier, P.M., and C.P. Keller. 1996. Multivariate interpolation to incorporate thematic surface data using inverse distance weighting (IDW). *Computers and Geosciences* 22: 795-799. https://doi.org/10.1016/0098-3004(96)00021-0

Batista, B.L., D. Grotto, M.F.H. Carneiro, and F. Barbosa. 2012. Evaluation of the Concentration of Nonessential and Essential Elements in Chicken, Pork, and Beef Samples Produced in Brazil. *Journal of Toxicology and Environmental Health, Part A* 75: 1269-1279. https://doi.org/10.1080/15287394.2012.709439

Bełdowska, M., A. Jędruch, L. Łęczyński, D. Saniewska, and U. Kwasigroch. 2016. Coastal erosion as a source of mercury into the marine environment along the Polish Baltic shore. *Environmental Science and Pollution Research* 23: 16375-16382. https://doi.org/10.1007/s11356-016-6753-7

Berzas Nevado, J.J., L.F. Garcia Bermejo, and R.C. Rodríguez Martín-Doimeadios. 2003. Distribution of mercury in the aquatic environmentat Almaden, Spain. *Environmental Pollution* 122: 261-271. https://doi.org/10.1016/S0269-7491(02)00290-7

Berzas Nevado, J.J., R.C. Rodríguez Martín-Doimeadios, F.J. Guzman Bernardo, M. Jimenez Moreno, S. Ortega Tardío, M.M. Sanchez-Herrera Fornieles, S. Martin-Nieto Ríos, and A. Doncel Perez. 2009. Integrated pollution evaluation of the Tagus River in Central Spain. *Environmental Monitoring and Assessment* 156: 461-477. https://doi.org/10.1007/s10661-008-0498-9

Berzas Nevado, J.J., R.C. Rodríguez Martín-Doimeadios, R. Mateo, N. Rodríguez Fariñas, J. Rodríguez-Estival, and M.J. Patiño Ropero. 2012. Mercury exposure and mechanism of response in large game using the Almadén mercury mining area (Spain) as a case study. *Environmental Research* 112: 58-66. https://doi.org/10.1016/j.envres.2011.09.019

Bilandžić, N., D. Dezdek, M. Sedak, M. Dokic, B. Solomun, I. Varenina, Z. Knezevic, and A. Slavica. 2010a. Concentrations of trace elements in tissues of red fox (*Vulpes vulpes*) and stone marten (*Martes foina*) from suburban and rural areas in Croatia. *Bulletin of Environmental Contamination and Toxicology* 85: 486-491. https://doi.org/10.1007/s00128-010-0146-2

Bilandžić, N., M. Sedak, M. Đokić, and B. Šimić. 2010b. Wild Boar Tissue Levels of Cadmium, Lead and Mercury in Seven Regions of Continental Croatia. *Bulletin of Environmental Contamination and Toxicology* 84: 738-743. https://doi.org/10.1007/s00128-010-9999-7

Bogans, E., Z. Gavare, A. Svagere, R. Poikane, and J. Skudra. 2011. Mercury Pollution Exploration in Latvia with High-Sensitivity Zeeman Atomic Absorption Spectrometry. *Environmental and Climate Technologies* 7: 39-45. https://doi.org/10.2478/v10145-011-0026-y

Bravo, A.G., D.N. Kothawala, K. Attermeyer, E. Tessier, P. Bodmar, J.L.J. Lefesma, J. Audet, J.P. Casas-Ruiz, et al. 2018. The interplay between total mercury, methylmercury and dissolved organic matter in fluvial systems: A latitudinal study across Europe. *Water Research* 144: 172-182. https://doi.org/10.1016/j.watres.2018.06.064

Čelechovská, O., L. Malota, and S. Zima. 2008. Entry of Heavy Metals into Food Chains: a 20-year Comparison Study in Northern Moravia (Czech Republic). *Acta Veterinaria Brno* 77: 645-652. https://doi.org/10.2754/avb200877040645

Champoux, L., J. Rodrigue, B. Braune, and D. Leclair. 1999. Contaminants in Northern Québec wildlife. In *Synopsis of research*

conducted under the 1997-1998 Northern Contaminants Program, ed. J. Jensen, 109-116. Ottawa: Department of Indian Affairs and Northern Development.

Chibunda, R.T., and C.R. Janssen. 2009. Mercury residues in free-grazing cattle and domestic fowl form the artisanal gold mining area of Geita district, Tanzania. *Food Additives and Contaminants, Part A* 26: 1482-1487. https://doi.org/10.1080/02652030903114928

Corsolini, S., S. Focardi, C. Leonzio, S. Lovari, F. Monaci, and G. Romeo. 1999. Heavy metals and chlorinated hydrocarbon concentrations in the red fox in relation to some biological parameters. *Environmental Monitoring and Assessment* 54: 87-100. https://doi.org/10.1023/A:1005974014029

Dainowski, B.H., L.K. Duffy, J. McIntyre, and P. Jones. 2015. Hair and bone as predictors of tissular mercury concentration in the Western Alaska Red Fox, *Vulpes vulpes*. *Science of The Total Environment* 518-519: 526-533. https://doi.org/10.1016/j.scitotenv.2015.03.013

Davis, D.D., J.R. McClenahen, and R.J. Hutnik. 2007. Use of the Moss *Dicranum montanum* to Evaluate Recent Temporal Trends of Mercury Accumulation in Oak Forests of Pennsylvania. *Northeastern Naturalist* 14: 27-34. https://doi.org/10.1656/1092-6194(2007)14[27:uotmdm]2.0.co;2

Demibraş, A. 1999. Proximate and heavy metal composition in chicken meat and tissues. *Food Chemistry* 67: 27-31.

Dobrowolska, A., and M. Melosik. 2002. Mercury contents in liver and kidneys of wild boar (*Sus scrofa*) and red deer (*Cervus elaphus*). *Zeitschrift fur Jagdwissenschaft* 48: 156-160.

Dobrzański, Z., R. Kołacz, S. Czaban, F. Bubel, M. Malczewski, R. Kupczyński, and S. Opalinski. 2017. Assessing Mercury Content in Plant and Animal Raw Materials in an Area Impacted by the Copper Industry. *Polish Journal of Environmental Studies* 26: 577-583. https://doi.org/10.15244/pjoes/66709

Drevnick, P.E., A.P. Roberts, R.R. Otter, C.R. Hammerschmidt, R. Klaper, and J.T. Oris. 2008. Mercury toxicity in livers of northern pike (*Esox lucius*) from Isle Royale, USA. *Comparative Biochemistry and Physiology, Part C* 147: 331-338. https://doi.org/10.1016/j.cbpc.2007.12.003

Durkalec, M., J. Szkoda, R. Kolacz, S. Opalinski, A. Nawrocka, and J. Zmudzki. 2015. Bioaccumulation of Lead, Cadmium and Mercury in Roe Deer and Wild Boars from Areas with Different Levels of Toxic Metal Pollution. *International Journal of Environmental Research* 9: 205-211. https://doi.org/10.22059/IJER.2015.890

Dušek, L., Z. Svobodová, D. Janoušková, B. Vykusová, J. Jarkovský, R. Šmíd, and P. Pavliš. 2005. Bioaccumulation of mercury in muscle tissue of fish in the Elbe River (Czech Republic): multispecies monitoring study 1991–1996. *Ecotoxicology and Environmental Safety* 61: 256-267. https://doi.org/10.1016/j.ecoenv.2004.11.007

EC. 2013. Directive 2013/39/EU of the European Parliament and of the Council of 12 August 2013 amending Directives 2000/60/EC and 2008/105/EC as regards priority substances in the field of water policy Text with EEA relevance. *OJ L* 226: 1-17. http://data.europa.eu/eli/dir/2013/39/oj

Edwards, S.C., C.L. MacLeod, and J.N. Lester. 1999. Mercury Contamination of the Eel (*Anguilla anguilla*) and Roach (*Rutilus rutilus*) in East Anglia, UK. *Environmental Monitoring Assessment* 55: 371-387. https://doi.org/10.1023/A:100593093

Egler, S.G., S. Rodrigues-Filho, R.C. Villas-Bôas, and C. Beinhoff. 2006. Evaluation of mercury pollution in cultivated and wild plants from two small communities of the Tapajós gold mining reserve, Pará State, Brazil. *Science of The Total Environment* 368: 424-433. https://doi.org/10.1016/j.scitotenv.2005.09.037

Eklöf, K., J. Fölster, L. Sonesten, and K. Bishop. 2012. Spatial and temporal variation of THg concentrations in run-off water from 19 boreal catchments, 2000-2010. *Environmental Pollution* 164: 102-109. https://doi.org/10.1016/j.envpol.2012.01.024

Elvince, R., T. Inoue, K. Tsushima, R. Takayanagi, U. Darung, S. Gumiri, S. Dohong, O. Nagafuchi, et al. 2008. Assessment of Mercury Contamination in the Kahayan River, Central Kalimantan, Indonesia. *Journal of Water and Environment Technology* 6: 103-112.

Ertl, K., R. Kitzer, and W. Goessler. 2016. Elemental composition of game meat from Austria. *Food Additives and Contaminants, Part B* 9: 120-126. https://doi.org/10.1080/19393210.2016.1151464

Falandysz, J. 1991. Manganese, Copper, Zinc, Iron, Cadmium, Mercury and Lead in Muscle Meat, Liver and Kidneys of Poultry, Rabbit and Sheep Slaughtered in the Northern Part of Poland, 1987. *Food Additives and Contaminants, Part B* 8: 71-83. https://doi.org/10.1080/02652039109373957

Falandysz, J. 1993. Some toxic and essential trace metals in swine from Northern Poland. *Science of The Total Environment* 136: 193-204. https://doi.org/10.1016/0048-9697(93)90307-R

Falandysz, J. 1994. Some toxic and trace metals in big game hunted in the northern part of Poland in 1987–1991. *Science of the Total Environment* 141: 59-73. https://doi.org/10.1016/0048-9697(94)90018-3

Falandysz, J., M. Gucia, A. Frankowska, M. Kawano, and B. Skwarzec. 2001. Total mercury in wild mushrooms and underlying soil substrate from the city of Umeå and its surroundings, Sweden. *Bulletin of Environmental Contamination and Toxicology* 20: 247-253. https://doi.org/10.1007/s001280188

Falandysz, J., A. Frankowska, and A. Mazur. 2007. Mercury and its bioconcentration factors in King Bolete (*Boletus edulis*) Bull. Fr. *Journal of Environmental Science and Health, Part A* 42: 2089-2095. https://doi.org/10.1080/10934520701627058

Falandysz, J., J. Zhang, Y.Z. Wang, M. Saba, G. Krasińska, A. Wiejak, and T. Li. 2015. Evaluation of Mercury Contamination in Fungi Boletus Species from Latosols, Lateritic Red Earths, and Red and Yellow Earths in the Circum-Pacific Mercuriferous Belt of Southwestern China. *PLoS ONE* 10: e0143608. https://doi.org/10.1371/journal.pone.0143608

Falter R., and H.F. Schöler. 1994. Determination of methyl-, ethyl-, phenyl and total mercury in Neckar river fish. *Chemosphere* 29: 1333-1338. https://doi.org/10.1016/0045-6535(94)90263-1

Farkas, A., J. Salanki, and A. Specziar. 2003. Age- and size-specific patterns of heavy metals in the organs of freshwater fish *Abramis brama* L. populating a low-contaminated site. *Water Research* 37: 959-964. https://doi.org/10.1016/S0043-1354(02)00447-5

Feng, X., G. Qiu, S. Wang, and L. Shang. 2003. Distribution and speciation of mercury in surface waters in mercury mining areas in Wanshan, Southwestern China. *Journal de Physique IV France* 107: 455-458. https://doi.org/10.1051/jp4:20030339

Fleit, E., and G. Lakatos. 2003. Accumulative heavy metal patterns in the sediment and biotic compartments of the Tisza watershed. *Toxicology Letters* 140-141: 323-332. https://doi.org/10.1016/s0378-4274(03)00029-8

Florijancic, T., S. Ozimec, D. Jelkic, N. Vuksic, N. Bilandzic, A. Gross Boskovic, and I. Boskovic. 2015. Assessment of heavy metal content in wild boar (*Sus scrofa* L.) hunted in eastern Croatia. *Journal of Environmental Protection and Ecology* 16: 630-636.

Ganguli, P.Y., R.P. Mason, K.E. Abu-Saba, R.S. Anderson, and A.R. Flegal. 2000. Mercury Speciation in Drainage from the New Idria Mercury Mine, California. *Environmental Science and Technology* 34: 4773-4779. https://doi.org/10.1021/es991364y

Gasparik, J., M. Dobias, M. Capcarova, P. Smehyl, J. Slamecka, J. Bujko, and J. Gasparik Jr. 2012. Concentration of cadmium, mercury, zinc, copper and cobalt in the tissues of wild boar (*Sus scrofa*) hunted in the western Slovakia. *Journal of Environmental Science and Health, Part A* 47: 1212-1216. https://doi.org/10.1080/10934529.2012.672065

Gębka, K., M. Bełdowska, D. Saniewska, K. Kuliński, and J. Bełdowski. 2018. Watershed characteristics and climate factors effect on the temporal variability of mercury in the southern Baltic Sea rivers. *Journal of Environmental Sciences* 68: 55-64. https://doi.org/10.1016/j.jes.2017.11.030

Gentès, S., R. Maury-Brachet, R. Guyneaud, M. Monperrus, J.M. André, S. Davail, and A. Legeay. 2013. Mercury bioaccumulation along food webs in temperate aquatic ecosystems colonized by aquatic macrophytes in south western France. *Ecotoxicology and Environmental Safety* 91: 180-187. https://doi.org/10.1016/j.ecoenv.2013.02.001

Ghimpeteanu, O.M., K. Das, M. Melitaru, and M.L. Scippo. 2012. Assessment of Heavy Metals and Mineral Nutrients in Poultry Liver Using Inductively Coupled Plasma-mass Spectrometer (ICP-MS) and Direct Mercury Analyzer (DMA). *Bulletin of the University of Agricultural Sciences and Veterinary* 69: 353-359.

Giżejewska, A., A. Spodniewska, and D. Barski. 2014. Concentration of lead, cadmium, and mercury in tissues of European beaver (*Castor fiber*) from the north-eastern Poland. *Bulletin of the Veterinary Institute in Pulawy* 58: 77-80. https://doi.org/10.2478/bvip-2014-0012

Gnamuš, A., A.R. Byrne, and M. Horvat. 2000. Mercury in the Soil-Plant-Deer-Predator Food Chain of a Temperate Forest in Slovenia. *Environmental Science and Technology* 34: 3337-3345. https://doi.org/10.1021/es991419w

Gomes Ribeiro, D.R., H. Faccin, T.R. Dal Molin, L.M. de Caralho, and L.L. Amado. 2017. Metal and metalloid distribution in different environmental compartments of the middle Xingu River in the Amazon, Brazil. *Science of The Total Environment* 605-606: 66-74. https://doi.org/10.1016/j.scitotenv.2017.06.143

Gray, J.E., P.M. Theodorakos, E.A. Bailey, and R.R. Turner. 2000. Distribution, speciation, and transport of mercury in stream-sediment, stream-water, and fish collected near abandoned mercury mines in southwestern Alaska, USA. *Science of The Total Environment* 260: 21-33. https://doi.org/10.1016/S0048-9697(00)00539-8

Gufler, H., F. Tataruch, and A.K. Onderscheka. 1997. Investigation of the lead, cadmium and mercury contents in the organs and muscles of roe deer and chamois in southern Tyrol. *Zeitschrift fur Jagdwissenschaft* 43: 240-250.

Harmens, H., D.A. Norris, G.R. Koerber, A. Buse, E. Steinnes, and Å. Rühling. 2008. Temporal trends (1990-2000) in the concentration of cadmium, lead and mercury in mosses across Europe. *Environmental Pollution* 151: 368-376. https://doi.org/10.1016/j.envpol.2007.06.043

Hashemi, M. 2018. Heavy metal concentrations in bovine tissues (muscle, liver and kidney) and their relationship with heavy metal contents in consumed feed. *Ecotoxicology and Environmental Safety* 154: 263-267. https://doi.org/10.1016/j.ecoenv.2018.02.058

Hattula, M.L., J. Sarkka, J. Janatuinen, J. Paasivirta, and A. Roos. 1978. Total mercury and methyl mercury contents in fish from lake Päijänne. *Environmental Pollution* 17: 19-29. https://doi.org/10.1016/0013-9327(78)90052-6

HELCOM. 2015. Guidelines for the annual and periodical compilation and reporting of waterborne pollution inputs to the Baltic Sea (PLC-Water). Helsinki: Baltic Marine Environment Protection Commission.

HELCOM. 2018. HELCOM metadata catalogue. Retrieved 23 September, 2020, from http://metadata.helcom.fi/geonetwork/srv/eng/catalog.search#/metadata/20be292d-e770-4242-8151-4fd4c35f461b

Heyes, A., C. Miller, and R.P. Mason. 2004. Mercury and methylmercury in Hudson River sediment: impact of tidal resuspension on partitioning and methylation. *Marine Chemistry* 90: 75-89. https://doi.org/10.1016/j.marchem.2004.03.011

Horvat, M., N. Nolde, V. Fajon, V. Jereb, M. Logar, S. Lojen, R. Jacimovic, I. Falnoga, et al. 2003. Total mercury, methylmercury and selenium in mercury polluted areas in the province Guizhou, China. *Science of The Total Environment* 304: 231-256. https://doi.org/10.1016/s0048-9697(02)00572-7

Jewett, S.C., X. Zhang, A. Sathy Naidu, J.J. Kelley, D. Dasher, and L.K. Duffy. 2003. Comparison of mercury and methylmercury in northern pike and Arctic grayling from western Alaska rivers. *Chemosphere* 50: 383-392. https://doi.org/10.1016/S0045-6535(02)00421-6

Jorhem, L., S. Slorach, B. Sundström, and B. Ohlin. 1991. Lead, cadmium, arsenic and mercury in meat, liver and kidney of Swedish pigs and cattle in 1984–88. *Food Additives and Contaminants* 8: 201-211. https://doi.org/10.1080/02652039109373970

Kalisińska, E., H. Budis, N. Lanocha, J. Podlasinska, E. Jedrzejewska, and D.I. Kosik-Bogacka. 2012a. Comparison of Hepatic and Nephric Total Mercury Concentrations Between Feral and Ranch American Mink (*Neovison vison*) from Northwestern Poland. *Bulletin of Environmental Contamination and Toxicology* 88: 802-806. https://doi.org/10.1007/s00128-012-0555-5

Kalisińska, E., P. Lisowski, and D.I. Kosik-Bogacka. 2012b. Red fox *Vulpes vulpes* (L., 1758) as a bioindicator of mercury contamination in terrestrial ecosystems of north-western Poland. *Biological Trace Element Research* 145: 172-180. https://doi.org/10.1007/s12011-011-9181-z

Kalisińska, E., N. Lanocha-Arendarczyk, D.I. Kosik-Bogacka, H. Budis, B. Pilarczyk, A. Tomza-Marciniak, J. Podlasinska, L. Cieslik, et al. 2017. Muscle mercury and selenium in fishes and semiaquatic

mammals from a selenium-deficient area. *Ecotoxicology and Environmental Safety* 136: 24-30. https://doi.org/10.1016/j.ecoenv.2016.10.028

Kambamanoli-Dimou, A., S. Kilikidis, and A. Kamarianos. 1989. Methylmercury concentrations in broiler's meat and hen's meat and eggs. *Bulletin of Environmental Contamination and Toxicology* 42: 728-734.

Kavčič, A., K. Mikuš, M. Debeljak, J.T. van Elteren, I. Arčon, A. Kodre, P. Kump, A.G. Karydas, et al. 2019. Localization, ligand environment, bioavailability and toxicity of mercury in *Boletus* spp. and *Scutiger pes-caprae* mushrooms. *Ecotoxicology and Environmental Safety* 184: 109623. https://doi.org/10.1016/j.ecoenv.2019.109623

Kensova, R., O. Celechovska, J. Doubravova, and Z. Svobodova. 2010. Concentrations of metals in tissues of fish from the Vestonice reservoir. *Acta Veterinaria Brno* 79: 335-345.

Khabarova, L.S., E.S. Ivanova, N.Y. Poddubnaya, A.V. Andreeva. A.P. Selezneva, and D.M. Feneva. 2018. Mercury in Tissues of Red Fox as Indicator of Environmental Pollution. *Advances in Engineering Research* 177: 96-100. https://doi.org/10.2991/isees-18.2018.19

Khallafalla, F.A., F.H. Ali, F. Schwagele, and M.A. Abd-El-Wahab. 2011. Heavy metal residues in beef carcasses in Beni-Suef abattoir, Egypt. *Veterinaria Italiana* 47: 351-361.

Kłos, A., M. Rajfur, I. Šrámek, and M. Wacławek. 2012. Mercury concentration in lichen, moss and soil samples collected from the forest areas of Praded and Glacensis Euroregions (Poland and Czech Republic). *Enviornmental Monitoring and Assessment* 184: 6765-6774. https://doi.org/10.1007/s10661-011-2456-1

KOBiZE. 2019. *Poland's Informative Inventory Report*. Warsaw: Institute of Environmental Protection – National Research Institute.

Komov, V.T., E.S. Ivanova, V.A. Gremyachikh, and N.Y. Poddubnaya. 2016. Mercury Content in Organs and Tissues of Indigenous (*Vulpes vulpes* L.) and Invasive (*Nyctereutes procyonoides* Gray.) Species of Canids from Areas Near Cherepovets (North-Western Industrial Region, Russia). *Bulletin of Environmental Contamination and Toxicology* 97: 480-485. https://doi.org/10.1007/s00128-016-1891-7

Koval, P.V., G.V. Kalmychkov, V.F. Gelety, G.A. Leonova, V.I. Medvedev, and L.D. Andrulaitis. 1999. Correlation of natural and technogenic mercury sources in the Baikal polygon, Russia. *Journal of Geochemical Exploration* 66: 277-289. https://doi.org/10.1016/S0375-6742(99)00041-2

Lavigne, M., M. Lucotte, and S. Paquet. 2010. Relationship between Mercury Concentration and Growth Rates for Walleyes, Northern Pike, and Lake Trout from Quebec Lakes. *North American Journal of Fisheries Management* 30: 1221-1237. http://dx.doi.org/10.1577/M08-065.1

Lawson, N.M., R.P. Mason, and J.M. Laprote. 2001. The fate and transport of mercury, methylmercury, and other trace metals in Chesapeake Bay tributaries. *Water Research* 35: 501-515. https://doi.org/10.1016/S0043-1354(00)00267-0

Lazarus, M., T. Orct, M. Blanuša, I. Vicković, and B. Šoštarić. 2008. Toxic and essential metal concentrations in four tissues of red deer (*Cervus elaphus*) from Baranja, Croatia. *Food Additives and Contaminants, Part A* 25: 270-283. https://doi.org/10.1080/02652030701364923

Lazarus, M., A.P. Crnić, N. Bilandžić, J. Kusak, and S. Reljić. 2014. Cadmium, lead, and mercury exposure assessment among Croatian consumers of free-living game. *Archives of Industrial Hygiene and Toxicology* 65: 281-291. https://doi.org/10.2478/10004-1254-65-2014-2527

Lehel, J., P. Laczay, A. Gyurscó, F. Jánoska, S. Majoros, K. Lányi, and M. Marosán. 2015. Toxic heavy metals in the muscle of roe deer (*Capreolus capreolus*) – food toxicological significance. *Environmental Science and Pollution Research* 23: 4465-4472. https://doi.org/10.1007/s11356-015-5658-1

Lepom, P., and J. Wellmitz. 2017. Mercury Monitoring in Fish under the WFD – A German Perspective. International Conference on Chemistry and the Environment, Oslo, 18-22 June 2017 (oral presentation).

Lindeström, L. 2001. Mercury in Sediment and Fish Communities of Lake Vänern, Sweden: Recovery from Contamination. *Ambio* 30: 538-544. https://doi.org/10.1579/0044-7447-30.8.538

Lino, A.S., D. Kasper, Y.S. Giuda. J.R. Thomaz, and O. Malm. 2019. Total and methyl mercury distribution in water, sediment, plankton and fish along the Tapajós River basin in the Brazilian Amazon. *Chemosphere* 235: 690-700. https://doi.org/10.1016/j.chemosphere.2019.06.212

Liu, J., X. Feng, W. Zhu, X. Zhang, and R. Yin. 2011. Spatial distribution and speciation of mercury and methyl mercury in the surface water of East River (Dongjiang) tributary of Pearl River Delta, South China. *Environmental Science and Pollution Research* 19: 105-112. https://doi.org/10.1007/s11356-011-0542-0

López-Alonso, M., M. Miranda, C. Castillo, J. Hernández, M. García-Vaquero, and J.L. Benedito. 2007. Toxic and essential metals in liver, kidney and muscle of pigs at slaughter in Galicia, north-west Spain. *Food Additives and Contaminants* 24: 943-954. https://doi.org/10.1080/02652030701216719

Łuczyńska, J., M.J. Łuczyński, B. Paszczyk, and E. Tońska. 2016. Concentration of mercury in muscles of predatory and non-predatory fish from lake Pluszne (Poland). *Journal of Veterinary Research* 60: 43-47. https://doi.org/10.1515/jvetres-2016-0007

Łuczyńska, J., B. Paszczyk, and M.J. Łuczyński. 2018. Fish as a bioindicator of heavy metals pollution in aquatic ecosystem of Pluszne Lake, Poland, and risk assessment for consumer's health. *Ecotoxicology and Environmental Safety* 153: 60-67. https://doi.org/10.1016/j.ecoenv.2018.01.057

Lukáčová, A., Ł. Binkowski, and J. Golian. 2014. Mercury concentration in meat products. *Maso International* 1: 43-47.

Mão de Ferro, A., A.M. Mota, and J. Canário. 2014. Pathways and speciation of mercury in the environmental compartments of Deception Island, Antarctica. *Chemosphere* 95: 227-233. https://doi.org/10.1016/j.chemosphere.2013.08.081

Mariam, I., S. Iqbal, and S.A. Nagra. 2004. Distribution of Some Trace and Macrominerals in Beef, Mutton and Poultry. *International Journal of Agriculture and Biology* 6: 816-820.

Marsálek, P., Z. Svobodová, T. Randák, and J. Švehla. 2005. Mercury and Methylmercury Contamination of Fish from the Skalka Reservoir: A Case Study. *Acta Veterinaria Brno* 74: 427-434. https://doi.org/10.2754/avb200574030427

Maurice-Bourgoin, L, I. Quiroga, J.L. Guyot, and O. Malm. 1999. Mercury Pollution in the Upper Beni River, Amazonian Basin: Bolivia. *Ambio* 28: 302-306. https://www.jstor.org/stable/4314900

Mazej, Z., S. Al Sayegh-Petkovšek, and B. Pokorny. 2010. Heavy metal concentrations in food chain of lake Velenjsko Jezero, Slovenia: an artificial lake from mining. *Archives of Environmental Contamination and Toxicology* 58: 998-1007. https://doi.org/10.1007/s00244-009-9417-5

Mazurkiewicz, N., and J. Podlasińska. 2014. The mercury content of macrofungi from area of west Pomeranian district. *Bromatologia i Chemia Toksykologiczna* 47: 114-119.

Mazzoni, M., M. Petracci, A. Meluzzi, C. Cavani, P. Clavenzani, and F. Sirri. 2015. Relationship between pectoralis major muscle histology and quality traits of chicken meat. *Poultry Science* 94: 123-130. https://doi.org/10.3382/ps/peu043

Meinelt, T., R. Krüger, M. Pietrock, R. Osten, and C. Steinberg. 1997. Mercury pollution and macrophage centres in pike (*Esox lucius*) tissues. *Environmental Science and Pollution Research* 4: 32-36. https://doi.org/10.1007/bf02986262

Melgar, M.J., J. Alonso, and M.A. Garcia. 2009. Mercury in edible mushrooms and underlying soil: Bioconcentration factors and toxicological risk. *Science of The Total Environment* 407: 5328-5334. https://doi.org/10.1016/j.scitotenv.2009.07.001

Migaszewski, Z.M., A. Gałuszka, S. Dołęgowska, J.G. Crock, and P.J. Lamothe. 2010. Mercury in mosses *Hylocomium splendens* (Hedw.) B.S.G. and *Pleurozium schreberi* (Brid.) Mitt. from Poland and Alaska: Understanding the origin of pollution sources. *Ecotoxicology and Environmental Safety* 73: 1345-1351. https://doi.org/10.1016/j.ecoenv.2010.06.015

Millán, J., R. Mateo, M.A. Taggart, J.V. Lopez-Bao, M. Viota, L. Monsalve, P.R. Camarero, E. Blazquez, et al. 2008. Levels of heavy metals and metalloids in critically endangered Iberian lynx and other wild carnivores from Southern Spain. *Science of The Total Environment* 399: 193-201. https://doi.org/10.1016/j.scitotenv.2008.03.038

Molina, J.A., R. Oyarzun, J.M. Esbri, and P. Higueras. 2006. Mercury accumulation in soils and plants in the Almaden mining district, Spain: one of the most contaminated sites on Earth. *Environmental Geochemistry and Health* 28: 487-498. https://doi.org/10.1007/s10653-006-9058-9

Musilová, J., P. Trebichalský, I. Jančo, T. Tóth, and M. Šnirc. 2019. Mercury bioaccumulation in *Boletus edulis* bull. In different forest ecosystems in Slovakia. *GeoConference SGEM* 19: 753-760. https://doi.org/10.5593/sgem2019/3.2

Naik, A.P., and C.R. Hammerschmidt. 2011. Mercury and trace metal partitioning and fluxes in suburban Southwest Ohio watersheds. *Water Research* 45: 5151-5160. https://doi.org/10.1016/j.watres.2011.07.023

Nawrocka, A., M. Durkalec, J. Szkoda, A. Filipek, M. Kmiecik, J. Żmudzki, and A. Posyniak. 2020. Total mercury levels in the muscle and liver of livestock and game animals in Poland, 2009–2018. *Chemosphere* 258: 127311. https://doi.org/10.1016/j.chemosphere.2020.127311

Nguetseng, R., A. Fliedner, B. Knopf, B. Lebreton, M. Quack, and H. Rüdel. 2015. Retrospective monitoring of mercury in fish from selected European freshwater and estuary sites. *Chemosphere* 134: 427-434. https://doi.org/10.1016/j.chemosphere.2015.04.094

Niemi, A., E.R. Venäläinen, T. Hirvi, J. Hirn, and E. Karppanen. 1991. The lead, cadmium and mercury concentrations in muscle, liver and kidney from Finnish pigs and cattle during 1987-1988. *Zeitschrift fur Jagdwissenschaft* 192: 427-429. https://doi.org/10.1007/BF01193141

Nkansah, M.A., and J.K. Ansah. 2014. Determination of Cd, Hg, As, Cr and Pb levels in meat from the Kumasi Central Abattoir. *International Journal of Scientific and Research Publications* 4: 61-64.

Noël, L., R. Chekri, S. Millour, M. Merlo, J.C. Leblanc, and T. Guérin. 2013. Distribution and relationships of As, Cd, Pb and Hg in freshwater fish from five French fishing areas. *Chemosphere* 90: 1900-1910. https://doi.org/10.1016/j.chemosphere.2012.10.015

Nogueira, N., N. Cordeiro, and M.J. Aveiro. 2013. Chemical composition, fatty acids profile and cholesterol content of commercialized marine fishes captured in Northeastern Atlantic. *Journal of Fisheries Science* 7: 271-286. https://doi.org/10.3153.jfscom.2013029

Olson, C.L., M. Jiskra, J.E. Sonke, and D. Obrist. 2019. Mercury in tundra vegetation of Alaska: Spatial and temporal dynamics and stable isotope patterns. *Science of The Total Environment* 660: 1502-1512. https://doi.org/10.1016/j.scitotenv.2019.01.058

Olsson, I.M., S. Jonsson, and A. Oskarsson. 2001. Cadmium and zinc in kidney, liver, muscle and mammary tissue from dairy cows in conventional and organic farming. *Journal of Environmental Monitoring* 3: 531-538. https://doi.org/10.1039/b104506g

Opaliński, S., Z. Dobrzański, P. Pokorny, and R. Barej. 2004. The research of concentration of mercury in tissues of laying hens housing in free range system in agricultural and industrial regions. *Zeszyty Naukowe Akademii Rolniczej we Wrocławiu* 501: 221-229.

Ovsepyan, A.E., Y.A. Fedorov, A.A. Zimovets, and V.A. Savitsky. 2016. Diurnal dynamics of mercury in water objects of the north European part of Russia. Diurnal dynamics of mercury in water objects of the north European part of Russia. *GeoConference SGEM* 1: 243-250.

Packer, B.N., G.T. Carling, T.J. Veverica, K.A. Russell, S.T. Nelson, Z.T. Aanderud. 2020. Mercury and dissolved organic matter dynamics during snowmelt runoff in a montane watershed, Provo River, Utah, USA. *Science of The Total Environment* 704: 135297. https://doi.org/10.1016/j.scitotenv.2019.135297

Parinet, J., E. Royer, M. Saint-Hilaire, C. Chafey, L. Noël, B. Minvelle, G. Dervilly-Pinel, E. Engel, et al. 2018. Classification of trace elements in tissues from organic and conventional French pig production. *Meat Science* 141: 28-35. https://doi.org/10.1016/j.meatsci.2018.02.008

Pasieczna, A. 2012. *Polish geochemical atlas*. Warsaw: Geological Publishing House.

Petrović, Z. 2007. Investigation of cadmium, mercury and arsenic accumulation in wild boar tissues from hunting areas in Serbia. *Biotechnology in Animal Husbandry* 23: 83-88. https://doi.org/10.2298/BAH0704083P

Pirrone, N., G.J. Keeler, and J.O. Nriagu. 1996. Regional differences in worldwide emissions of mercury to the atmosphere. *Atmospheric Environment* 30: 2981-2987. https://doi.org/10.1016/1352-2310(95)00498-x

Piskorova, L., Z. Vasilkova, and I. Krupicer. 2003. Heavy metals residues in tissues of wild boar (*Sus scrofa*) and red fox (*Vulpes vulpes*) in the Central Zemplin region of the Slovak Republic. *Czech Journal of Animal Science* 48: 134-138.

Polak-Juszczak, L., and T. Nermer. 2016. Methylmercury and Total Mercury in Eels, Anguilla anguilla, from Lakes in Northeastern Poland: Health Risk Assessment. *EcoHealth* 13: 582-590. https://doi.org/10.1007/s10393-016-1139-3

Qiu, G., X. Feng, S. Wang, and L. Shang. 2005. Mercury and methylmercury in riparian soil, sediments, mine-waste calcines, and moss from abandoned Hg mines in east Guizhou province, southwestern China. *Applied Geochemistry* 20: 627-638. https://doi.org/10.1016/j.apgeochem.2004.09.006

Rempel, K. 2010. *What Is the Level of Mercury in Plants That Form the Diet of the Porcupine Caribou?* Ottawa: The Royal Canadian Geographical Society.

Rencz, A.N., N.J. O'Driscoll, G.E.M. Hall, T. Peron, K. Telmer, and N.M. Burgess. 2003. Spatial variation and correlations of mercury levels In the terrestrial and aquatic components of a Wetland dominated ecosystem: Kejimkujik Park, Nova Scotia, Canada. *Water, Air, and Soil Pollution* 143: 271-288. https://doi.org/10.1023/A:1022899919151

Riget, F., G. Asmund, and P. Aastrup. 2000. The use of lichen (*Cetraria nivalis*) and moss (*Rhacomitrium lanuginosum*) as monitors for atmospheric deposition in Greenland. *Science of The Total Environment* 245: 137-148. https://doi.org/10.1016/s0048-9697(99)00439-8

Rudy, M. 2009. The analysis of correlations between the age and the level of bioaccumulation of heavy metals in tissues and the chemical composition of sheep meat from the region in SE Poland. *Food and Chemical Toxicology* 47: 1117-1122. https://doi.org/10.1016/j.fct.2009.01.035

Saniewska, D. 2013. Input pathways of mercury to the coastal zone of the Gulf of Gdansk. PhD Thesis. Gdynia, Poland: University of Gdańsk.

Saniewska, D., M. Bełdowska, J. Bełdowski, A. Jędruch, M. Saniewski, and L. Falkowska. 2014a. Mercury loads into the sea associated with extreme flood. *Environmental Pollution* 191: 93-100. https://doi.org/10.1016/j.envpol.2014.04.003

Saniewska, D., M. Bełdowska, J. Bełdowski, M. Saniewski, M. Szubska, A. Romanowski, and L. Falkowska. 2014b. The impact of land use and season on the riverine transport of mercury into the marine coastal zone. *Environmental Monitoring and Assessment* 186: 7593-7604. https://doi.org/10.1007/s10661-014-3950-z

Scharenberg, W., P. Gramann, and W.H. Pfeiffer. 1994. Bioaccumulation of heavy metals and organochlorines in a lake ecosystem with special reference to bream (*Abramis brama* L.). *Science of The Total Environment* 155: 187-197. https://doi.org/10.1016/0048-9697(94)90291-7

Schuster, P.F., R.G. Striegl, G.R. Aiken, D.P. Krabbenhof, J.F. Dewild, K. Butler, B. Kamark, and M. Dornblaser. 2011. Mercury Export from the Yukon River Basin and Potential Response to a Changing Climate. *Environmental Science and Technology* 45: 9262-9267. https://doi.org/10.1021/es202068b

Shah, A.Q., T.G. Kazi, J.A. Baig, H.I. Afridi, G.A. Kandhro, M.B. Arain, N.F. Kolachi, and S.K. Wadhwa. 2010. Total mercury determination in different tissues of broiler chicken by using cloud point extraction and cold vapor atomic absorption spectrometry. *Food and Chemical Toxicology* 48: 65-69. https://doi.org/10.1016/j.fct.2009.09.016

Shao, J., C. Liu, Q. Zhang, J. Fu, R. Yang, J. Shi, Y. Cai, and G. Jiang. 2017. Characterization and speciation of mercury in mosses and lichens from the high-altitude Tibetan Plateau. *Environmental Geochemistry and Health* 39: 475. https://doi.org/10.1007/s10653-016-9828-y

Sheffy, T.B., and J.R.S. Amant. 1982. Mercury burdens in furbearers in Wisconsin. *Journal of Wildlife Management* 46: 1117-1120. https://doi.org/10.2307/3808255

Širić, I., P. Žurha, D. Barkić, and M.M. Staver. 2015. Trace Element Contents in the Edible Mushroom *Boletus edulis* Bull. ex Fries. *Agriculturae Conspectus Scientificus* 80: 223-227.

Sobańska, M.A. 2005. Wild boar hair (*Sus scrofa*) as a non-invasive indicator of mercury pollution. *Science of The Total Environment* 339: 81-88. https://doi.org/10.1016/j.scitotenv.2004.07.018

Sonesten, S. 2001. Mercury content in roach (*Rutilus rutilus* L.) in circumneutral lakes – effects of catchment area and water chemistry. *Environmental Pollution* 112: 471-481. https://doi.org/10.1016/S0269-7491(00)00135-4

Srebočan, E., A. Prevendar Crnić, A.M. Ekert-Kabalin, M. Lazarus, J. Jurasović, K. Tomljanović, D. Andreić, I. Strunjak Perović, et al. 2011. Cadmium, lead, and mercury concentrations in tissues of roe deer (*Capreolus capreolus* L.) and wild boar (*Sus scrofa* L.) from lowland Croatia. *Czech Journal of Food Sciences* 29: 624-633.

Suchara, I., J. Sucharova, M. Hola, C. Reimann, R. Boyd, P. Filzmoser, and P. Englmaier. 2011. The performance of moss, grass, and 1- and 2-year old spruce needles as bioindicators of contamination: A comparative study at the scale of the Czech Republic. *Science of The Total Environment* 409: 2281-2297. https://doi.org/10.1016/j.scitotenv.2011.02.003

Sun, X., Q. Zhang, M. Li, K. Kandel, B. Rawat, A. Pandey, J. Guo, S. Kang, et al. 2020. Mercury variation and export in trans-Himalayan rivers: Insights from field observations in the Koshi River. *Science of The Total Environment* 738: 139836. https://doi.org/10.1016/j.scitotenv.2020.139836

Surtipanti, S., S. Suwirma, S. Yumiarti, and Y. Mellawati. 1995. Determination of heavy metals in meat, intestine, liver, eggs, and chicken using neutron activation analysis and atomic absorption spectrometry. *Atom Indonesia* 21: 1-8.

Szkoda, J., and J. Żmudzki. 2001. Toxic elements in tissues of game animals. *Medycyna Weterynaryjna* 57: 883-886.

Szkoda, J., J. Żmudzki, A. Nawrocka, and M. Kmiecik. 2014. Toxic elements in free-living freshwater fish, water and sediments in Poland. *Bulletin of the Veterinary Institute in Pulawy* 58: 589-595. https://doi.org/10.2478/bvip-2014-0091

Terán-Mita, T.A., A. Faz, F. Salvador, J.M. Arocena, and J.A. Acosta. 2013. High altitude artisanal small-scale gold mines are hot spots for Mercury in soils and plants. *Environmental Pollution* 173: 103-109. https://doi.org/10.1016/j.envpol.2012.10.008

Vos, G., J.J.M.H. Teeuwen, and W. van Delft. 1986. Arsen, Cadmium, Blei und Quecksilber in Fleisch, Leber und Niere von Mastschweinen

in den Niederlanden während der Jahre 1980-1985. *Zeitschrift für Lebensmittel-Untersuchung und Forschung* 183: 397-401. https://doi.org/10.1007/BF01140547

Vos, G., J.P.C. Hovens, and W. van Delft. 1987. Arsenic, cadmium, lead and mercury in meat, livers and kidneys of cattle slaughtered in the Netherlands during 1980-1985. *Food Additives and Contaminants* 4: 73-88. https://doi.org/10.1080/02652038709373617

Wang, S., Y. Jia, S. Wang, X. Wang, H. Wang, Z. Zhao, and B. Liu. 2009. Total mercury and monomethylmercury in water, sediments, and hydrophytes from the rivers, estuary, and bay along the Bohai Sea coast, northeastern China. *Applied Geochemistry* 24: 1702-1711. https://doi.org/10.1016/j.apgeochem.2009.04.037

Wang, X., W. Yuan, X. Feng, D. Wang, and J. Luo. 2019. Moss facilitating mercury, lead and cadmium enhanced accumulation in organic soils over glacial erratic at Mt. Gongga, China. *Environmental Pollution* 254: 112974. https://doi.org/10.1016/j.envpol.2019.112974

Wu, J.J. 2014. Evaluation of Mercury Concentration in Yangtze River Estuary. *Journal of Anhui Agricultural Sciences* 19: 6339-6340.

Wyrzykowska, B., J. Falandysz, and G. Jarzyńska. 2012. Metals in edible fish from Vistula River and Dead Vistula River channel, Baltic Sea. *Journal of Environmental Science and Health, Part B* 47: 296-305. https://doi.org/10.1080/03601234.2012.638890

Xiao, Z., J. Sommar, O. Lindqvist, and E. Giouleka. 1998. Atmospheric mercury deposition to grass in southern Sweden. *Science of The Total Environment* 213: 85-94. https://doi.org/10.1016/s0048-9697(98)00080-1

Yamaguchi, N., D. Gazzard, G. Scholey, and D.W. Macdonald. 2003. Concentrations and hazard assessment of PCBs, organochlorine pesticides and mercury in fish species from the upper Thames: River pollution and its potential effects on top predators. *Chemosphere* 50: 265-273. https://doi.org/10.1016/S0045-6535(02)00482-4

Zamani-Ahmadmahmoodi, R., A.R. Bakhtiari, and J.A. Rodríguez Martín. 2014. Spatial relations of mercury contents in Pike (*Esox lucius*) and sediments concentration of the Anzali wetland, along the southern shores of the Caspian Sea, Iran. *Marine Pollution Bulletin* 84: 97-103. https://doi.org/10.1016/j.marpolbul.2014.05.030

Żarski, T.P., B. Debski, E. Rokicki, M. Samek, J. Valka, and I. Beseda. 1995a. Free living animals as bioindicators of mercury pollution. *Ekologia Bratislava* 14: 113-117.

Żarski, T.P., B. Debski, and M. Samek. 1995b. Relation between selenium and mercury concentrations in tissues of hares (*Lepus europaeus* Pall.) from regions with various environmental contaminations. *Ekologia Bratislava* 14: 93-97.

Zhang, D., A. Frankowska, G. Jarzyńska, A.K. Kojta, M. Drewnowska, D. Wydmańska, L. Bielawski, J. Wang, et al. 2010. Metals of King Bolete (*Boletus edulis*) Bull.: Fr. collected at the same site over two years. *African Journal of Agricultural Research* 5: 3050-3055.

Zięba, M. 2003. Concentrations of heavy metals in tissues of sheep from south-eastern Poland. *Przegląd Hodowlany* 6: 21-24.

Zolfaghari, G. 2018. Risk assessment of mercury and lead in fishspecies from Iranian international wetlands. *MethodsX* 5: 438-447. https://doi.org/10.1016/j.mex.2018.05.002
